# Assessing the heat sensitivity of Urdbean (*Vigna mungo* L. Hepper) genotypes involving physiological, reproductive and yield traits under field and controlled environment

**DOI:** 10.3389/fpls.2022.1042999

**Published:** 2022-11-21

**Authors:** Shikha Chaudhary, Uday Chand Jha, Pronob J. Paul, P. V. Vara Prasad, Kamal Dev Sharma, Sanjeev Kumar, Debjyoti Sen Gupta, Parul Sharma, Sarvjeet Singh, Kadambot H. M. Siddique, Harsh Nayyar

**Affiliations:** ^1^ Department of Botany, Panjab University, Chandigarh, India; ^2^ Crop Improvement Division, Indian Council of Agricultural Research (ICAR)-Indian Institute of Pulses Research, Kanpur, India; ^3^ International Rice Research Institute, South-Asia Hub, Hyderabad, India; ^4^ Sustainable Intensification Innovation Lab, Kansas State University, Manhattan, KS, United States; ^5^ Department of Agricultural Biotechnology, Choudhary Sarwan Kumar (CSK) Himachal Pradesh Agricultural University, Palampur, India; ^6^ Department of Plant Sciences, Central University of Punjab, Bhatinda, India; ^7^ Department of Plant Breeding and Genetics, Punjab Agricultural University, Ludhiana, India; ^8^ The University of Western Australia (UWA) Institute of Agriculture, The University of Western Australia, Perth, WA, Australia

**Keywords:** urdbean, heat stress, genetic variability, physiological traits, biochemical traits

## Abstract

The rising temperatures are seriously impacting the food crops, including urdbean; hence efforts are needed to identify the sources of heat tolerance in such crops to ensure global food security. In the present study, urdbean genotypes were evaluated for heat tolerance under natural outdoor for two consecutive years (2018, 2019) and subsequently in the controlled environment of the growth chamber to identify high temperature tolerant lines. The genotypes were assessed involving few physiological traits (membrane damage, chlorophyll, photosynthetic efficiency, stomatal conductance, lipid peroxidation), reproductive traits (pollen germination % and pollen viability %) and yield related traits (total number of pods plant^-1^, total seeds plant^-1^, single seed weight and seed yield plant^-1^). Based upon these tested traits, PantU31, Mash114, UTTARA and IPU18-04 genotypes were identified as promising genotypes for both years under heat stress condition. Further confirming heat tolerance, all these four tolerant and four sensitive genotypes were tested under controlled environment under growth chamber condition. All these four genotypes PantU31, Mash114, UTTARA and IPU18-04 showed high chlorophyll content, photosynthetic efficiency, stomatal conductance, leaf area, pods plant^-1^, total seeds plant^-1^ and low reduction in pollen germination % and pollen viability under stress heat stress condition. Moreover, yield and yield related traits viz., pods plant^-1^, seeds plant^-1^, single seed weight and seed yield plant^-1^ showed very strong positive correlation with pollen germination and pollen viability except electrolyte leakage and malondialdehyde content. Thus, these genotypes could be potentially used as donors for transferring heat tolerance trait to the elite yet heat-sensitive urdbean cultivars.

## 1 Introduction

Due to global climate change projections, heat waves are predicted to expand in many regions of the world imposing a huge threat to the agricultural security (Riaz et al., 2021). Variability around the optimum temperature is going to surge that will affect the complete life cycle or phenology of the plant (Chaturvedi et al., 2021). Continuously rising temperature (heat stress) has wide range of impacts on the overall morphology, anatomy and physiology of the plants (Chen et al., 2014). At sub-cellular levels, these impacts can be assessed using various biochemical and molecular approaches. Although heat stress has the potential to affect all the stages of plant but some stages are more vulnerable to heat stress; reproductive or seed filling stages are highly affected due to heat stress (Allakhverdiev et al., 2008; Hedhly, 2011). Moreover, the effects of high temperature are plant species- and stage- specific, the severity further depends on the duration and intensity of stress (Li et al., 2018).Various reports have suggested that heat stress disturbs the morphology of the plant by reducing its plant height, leaf area and root architecture (Chaudhary et al., 2020). At the cellular level, heat stress leads to protein denaturation, enzyme inactivation, membrane damage, exaggerate ROS generation, loss in water status and cellular viability. In leaves, photosynthetic machinery is reported as most sensitive to high temperature (Wahid et al., 2007; Bita and Gerats, 2013). Loss of chlorophyll content, denaturation of D1 protein of photosystem II and reduced carbon metabolism are mainly responsible for reducing photosynthetic rate (Allakhverdiev et al., 2008).Of the reproductive organ development stages; male gametophyte development and seed filling processes are reported to be extremely sensitive to even a few degree rise in temperature that can result in substantial yield loss (Hedhly, 2011). Impaired transport of sucrose to the developing reproductive organs under heat stress may restrict the flower development that brings out more flower abortion, pod abortion and shrivelled seeds (Bhandari et al., 2016). Plant responds to such damages by reprogramming and activating various mechanisms related to production of antioxidants, phytohormones, osmolytes, primary and secondary metabolites to ensure their survival (Chebrolu et al., 2016; Sharma et al., 2016; Jha et al., 2022). Therefore, correlation of crop phenology with temperature fluctuations is crucial for the better understanding of the impacts and defence strategies employed by plants for its adaptation.

Urdbean (*Vigna* *mungo* L. Hepper) is an important summer season food legume, cultivated mostly in many tropical and sub-tropical countries of Asia, Africa, America, and Australia (Joshi and Rao, 2017).Optimum temperature for its proper growth and development is 25-35°C and being a temperature sensitive crop, its yield is drastically reduced under high temperature exceeding 35°C (Anitha et al., 2016; Sen Gupta et al., 2021).Very little reports are available about the heat stress impacts as well as defence responses, especially at reproductive stage of this crop (Sen Gupta et al., 2021). It is vital to identify and characterise heat tolerant urdbean genotypes as well as to find out some leaf and pollen-based traits and mechanisms underlying heat tolerance. Heat tolerant urdbean genotypes can increase the cultivation of this food legume in summer season as well at warmer locations to extend its cultivation status. Thus, the aim of the present study was to screen selective number of genotypes of urdbean to heat stress in 2 successive years under outdoor environment to identify heat tolerant genotypes, followed by their validation and characterisation under controlled environment of the growth chamber involving some leaf and pollen-based traits.

## 2 Methodology

### 2.1 Field and growth chamber experiments

Urdbean genotypes (26) were procured from Indian Institute of Pulse Research, Kanpur, India and Punjab Agricultural University, Ludhiana, India (**Supplementary Table S1**). These genotypes were assessed for their heat tolerance under outdoor environment and controlled conditions of the growth chamber at the Department of Botany, Panjab University, Chandigarh, India. Urdbean seeds were raised in pots (8L capacity) containing a mixture of soil, sand, farmyard manure [2:1:1 (v/v)] and Tri-calcium phosphate fertilizer 10 mg kg^-1^. Seeds were soaked in distilled water overnight (12 h) and subsequently inoculated with suitable strain of *Rhizobia* before sowing. There were 5 pots genotype^-1^ and each pot had 5 seeds that were thinned to 3 plants pot^-1^ after emergence for their proper growth. Plants were fully irrigated daily (twice; morning and evening) to avoid any water paucity. Plants were arranged in a randomized complete block design. Meteorological data (daily temperature and relative humidity) from date of sowing to harvesting was recorded throughout the entire cropping season (**Supplementary Figure S1**). To evaluate the effects of heat stress against the control temperature, crop was sown twice during cropping season and for two subsequent years (Summers of 2018, 2019) (i) during the normal conditions (control), in the last week of March 2018, when the day/night temperatures (<35/25°C) were optimum for the plant’s growth and ii) in the last week of April, to expose the plants to heat stress (>40/30°C). (**Supplementary Figure S1**, **Supplementary Table S2**). The plants after harvest were recorded for number of pods, seeds, seed yield plant^-1^ and single seed weight.

For validation of the results, a subsequent study was conducted in the growth chamber under the controlled conditions on some selected contrasting genotypes (4 heat-tolerant and 4 heat- sensitive genotypes, 5 pots genotype^-1^ having 2 plants pot^-1^). These plants were initially raised in the outdoor natural environment to achieve full vegetative growth (Average temperature<35/25°C; average RH 61/41%; Max/min) and were subsequently transferred to growth chamber at the onset of bud stage for further analysis. To avoid any kind of heat shock situation, temperature was gradually raised (2°C per day) up to 42/32°C. The plants were maintained at this temperature up to maturity. Simultaneously, the control plants were maintained at 35/25°C.

After 10d of heat stress, fully expanded leaves at 2^nd^ and 3^rd^ positions from the topmost youngest leaf were from control and heat-stressed environments were evaluated for various physiological traits *viz.* SPAD chlorophyll content, chlorophyll fluorescence (Fv/Fm), electrolyte leakage (to assess membrane damage), stomatal conductance, leaf area, relative leaf water content, and malondialdehyde (MDA). The reproductive traits (pollen viability and pollen germination) were tested from flowers after 5d exposure to heat stress. All these traits were further correlated with yield traits like total number of pods plant^-1^, total number of seeds plant^-1^, total seed yield plant^-1^, single seed weight.

### 2.2 Physiological, reproductive and yield traits

To assess the effects of heat stress on the plant growth and yield, various traits were studied; data were taken from three plants in triplicates genotype^-1^, pooled and averaged. Mean values of replicates are presented through tables and figures.

### 2.3 Physiological traits

#### 2.3.1 Chlorophyll content

Chlorophyll content (SPAD value) was measured using Apogee-SPAD meter and its readings were taken between 10.00 and 11.00 h of a fully expanded tagged leaf on alternative days at full vegetative and reproductive stage from 30 DAS (days after sowing) (Devi et al., 2022).

#### 2.3.2 Chlorophyll fluorescence

PS II activity/stability or photosynthetic efficiency was measured as chlorophyll fluorescence. Readings were taken between 10.00-11.00 h of a fully expanded leaf using the dark adapted test of modulated chlorophyll fluorometer OS1-FL (Opti-Sciences, Tyngsboro, MA, United States) (Sharma et al., 2016).

#### 2.3.3 Electrolyte leakage

Stress injury to leaves was measured as electrolyte leakage. Fresh leaves (1.0 g) were collected and washed three times with deionised water to remove surface adhering electrolytes. Plant tissue was placed in closed vials containing 10 ml of deionised water and incubated it for 25°C on a rotary shaker for 24 h; the electrical conductivity of the solution (L_1_) was checked using a conductivity meter (ELICO CM 180, Hyderabad, India). Then the final conductivity (L_2_) was measured after heating samples in a water bath at 120°C for 20 min (Lutts et al., 1996). Electrolyte leakage was calculated as (L1/L2) × 100. The electrolyte leakage was expressed as electrical conductivity in µmhos g^–1^ DW.

#### 2.3.4 Stomatal conductance

Stomatal conductance was measured from a fully expanded leaf using a portable leaf porometer (model SC1 Decagon Devices, Pullman, WA, United States) at 11.00 h and was expressed as m molm^-2^s^-1^ (Awasthi et al., 2014).

#### 2.3.5 Leaf area

Area of fully expanded tagged leaves was determined using a measurement scale and multiplied with a ‘leaf factor’ (method derived from urdbean from the ratio of actual and measured leaf area of many types of leaves from top to bottom of a plant) (Sharma et al., 2016).

#### 2.3.6 Relative leaf water content

RLWC was measured by the method of Barrs and Weatherley (1962). Fresh leaves were collected and were washed three times to remove any kind of debris. After drying with blotters, they were weighed (fresh weight, FW) and then floated in the distilled water in a petri dish. After 2 h, leaves were taken out of petri dish, reweighed and surface dried with blotters. Leaves were then oven-dried at 110°C for 24h and again weighed for dry weight (DW). Final values for relative leaf water content was calculated as (FW-DW)/(TW-DW) × 100.

#### 2.3.7 Malondialdehyde content

Lipid peroxidation of the cell membrane was measured as malondialdehyde (MDA) content (Heath and Packer, 1968). One hundred mg fresh leaf tissue was extracted in 10 mL of 0.1% trichloroacetic acid (TCA). The homogenate was centrifuged at 15,000 rpm for 5 min. Supernatant was used as extract. Afterward, 4 mL of 0.5% thiobarbituric acid (in 20% trichloroacetic acid) was added to a 1-ml of the supernatant. This mixture was heated at 95°C for 30 minutes followed by immediate cooling in ice bath. Re-centrifugation of this mixture was performed again at 10,000 rpm for 10 min and the absorbance of the supernatant was taken at 532 nm. Values were expressed as nmol g^-1^ DW.

### 2.4 Reproductive traits

For evaluating reproductive function, flowers were collected 5 days after exposure to heat stress and assessed for following traits.

#### 2.4.1 Pollen germination

For testing pollen germination, pollen grain samples were taken in three replicates and each replicate consisted of five flowers genotype^-1^ (Brewbaker and Kwack, 1963). Pollen grains were collected and immersed in few drops of pollen germinating medium (10% sucrose, 990 mM potassium nitrate (pH 6.5), 1.64 mM boric acid, 812 mM magnesium sulphate and 1.3 mM calcium nitrate) (Kaushal et al., 2013).

#### 2.4.2 Pollen viability

Around 100 pollen grains were tested for the pollen viability with 0.5% acetocarmine/Alexander stain per genotype in three replicates. Selection of viable pollen grains was made on the basis of size (fully expanded), shape (triangular or spherical) and concentration of stain taken by them. Pollen grains were collected from freshly opened flowers and were pooled and checked for their viability (Kaushal et al., 2013).

### 2.5 Yield traits

For obtaining yield data, three plants genotype^-1^ in three replications (9 plants genotype^-1^) were harvested at maturity, wrapped in paper bags and dried in an oven at 65°C for at least three days. After drying, the total number of pods and seeds, total seed weight and single seed weight plant^-1^ were calculated (Sharma et al., 2016).

### 2.6 Statistical analyses

Urdbean plants were grown in outdoor environment for 2 consecutive years as well as under controlled environment of the growth chamber using RCBD. The analysis of data for computing standard errors and least significant differences (*P*<0.05*)* was performed using 2-factorial (temperature × genotypes) design using OPSTAT statistical software (CCS, HAU, Hisar, India). Genotypic correlation, heritability were analysed by using GenStat 15 software. The Euclidean dissimilarity matrix was constructed involving all the genotypes and traits, and were clustered using Ward’s method (Patterson and Thompson, 1971). The principal component analysis was done using the R package factoextra, and heat map analysis was performed according to Babicki et al. (2016).

## 3 Results

### 3.1 Physiological traits

#### 3.1.1 Electrolyte leakage

Electrolyte leakage (EL%) is one of the important physiological traits measuring membrane damage used for screening heat stress tolerant genotypes in plants. Heat stress significantly (P<0.01) damaged the membranes (**Supplementary Figure S2**, **Supplementary Table S3**). EL increased by 49 and 51% in heat-stressed plants, compared to controls, in the first and second years, respectively. Based on this trait, Mash 114 (18.5%, 17.73%), PantU31(21.77%, 20.8%), UTTARA (21.43%, 20.73%), IPU18-04 (18.17%, 19.73%) genotypes revealed low value for EL% under heat stress environment for both years. However, the genotypes IPU 18-6 (25.13%, 26.9%), Mash 218 (26.47%, 26%), SuG1153 (26.23%, 26.9%) exhibited high value for EL% under heat stress environment for both years suggesting their heat stress sensitivity. The high heritability values (82.6% and 86.85, for first and second years, respectively) for this trait were noted under heat stress see **Table 1**.

**Table 1:** General statistics of various traits in urdbean genotypes under heat stress environment.

	Heritability	CV%	Mean	Range
Heat stress 1^st^ year				
Chlorophyll content	85.5	8.1	15.1	11.4-19.8
chlorophyll fluorescence	90.1	5.3	0.53	0.41-0.61
Electrolyte leakage%	82.6	7.3	24.2	18.2-28
Leaf area	83.7	6.6	18.4	13.9-21.4
Stomatal conductance	96.5	8.1	28.9	22.3-45.6
Pods plant^-1^	98.5	13.8	5.72	2.4-15
Seeds plant^-1^	99.4	11.2	21.03	6.2-62
Seed yield plant^-1^	99.2	19	0.64	0.11-2.7
Single seed weight	92.2	12.1	0.03	0.02-0.04
Heat stress 2^nd^ year				
Chlorophyll content	92.7	7.6	15.7	12.3-21.3
Chlorophyll fluorescence	97.3	2.9	0.54	0.44-0.66
Electrolyte leakage%	86.8	6.7	24.4	17.3-27.7
Leaf area	89.8	6.1	17.6	14.7-20.6
Pods plant^-1^	99	11.3	6.16	2.8-15.9
Stomatal conductance	95.2	8.6	30	25.5-46.5
Seeds plant^-1^	99.6	9.4	22	5.3-71
Seed yield plant^-1^	99.6	15	0.73	0.16-2.03
Single seed weight	94.5	10.1	0.03	0.02-0.04
Growth chamber heat stress				
Chlorophyll content	94.5	7.5	16.35	13.27-19.67
chlorophyll fluorescence	98.8	3.7	0.54	0.42-0.66
Electrolyte leakage%	96.2	4.5	24.5	20.27-28.03
Leaf area	94.5	7.5	16.35	13.27- 19.67
Malondialdehyde	98.8	5.3	27.75	19.3-33.97
Pollen germination %	99.4	6.8	36.7	15.3-54.17
Pollen viability%	98.8	6.7	43.24	24.5-61.20
Relative water content	99.3	2.9	63.66	49.57- 79.1
Stomatal conductance	99.1	5.5	25.03	14.37-33.7
Seeds plant^-1^	99.4	9.1	29	4-52
Seed yield plant^-1^	99.5	10.6	1.26	0.15- 2.38
Single seed weight	98.7	7.4	0.03	0.02-0.04
Pods plant^-1^	99.4	9.1	8.79	2.7-14.73

#### 3.1.2 Stomatal conductance

Stomatal conductance (gS) varied significantly (P<0.01) across the genotypes in plants exposed to high temperature (**Supplementary Figure S3**, **Supplementary Table S3**). As a result of high temperature, gS decreased by 12 and 15% over control in 1^st^ and 2^nd^ year, respectively. Under heat stress environment, Mash 114 (45.6, 40.47) and Pant G 31(43.9, 46.53) genotypes showed high value for stomatal conductance in both years. Regarding heritability for gS, 96.5% (during the first year) and 95.2% (during second year) heritability values were noted (**Table 1**).

#### 3.1.3 Chlorophyll content

A significant genetic variation (P<0.01) was noticed in chlorophyll content among the genotypes under heat stress (**Supplementary Figure S4**, **Supplementary Table S3**). The range of leaf chlorophyll content was noted to be 11.4-19.8 mg g^-1^ FW during the first year and 12.3- 21.3 mg g^-1^ FW during second year under heat stress environment. An average reduction of 22 and 30% was observed due to heat stress, relative to controls, in 1^st^ and 2^nd^ year, respectively. High value for chlorophyll content was observed in Mash 114 (19.67, 18.97), PantG31 (19.77, 20.47), UTTARA (17, 20.4) genotypes under heat stress environment for both the years. This trait also showed high heritability (85.5% and 92.7% for 1^st^ and 2^nd^ years, respectively) and could be vital for selecting heat tolerant urdbean lines (**Table 1**).

#### 3.1.4 Chlorophyll fluorescence

Significant genetic variability for chlorophyll fluorescence (ChlF) (Fv/Fm) was noted under heat stress environment (P<0.01) (**Supplementary Figure S5**, **Supplementary Table S3**). Mean value for Fv/Fm was noted to be 0.53 during the first year and 0.54 during second year under heat stress environment. Heat stress caused about 28% reduction over control in both the years. The genotypes Mash 114 (0.61, 0.66), PantU31(0.61, 0.65), UTTARA (0.6,0.65) showed high value for ChlF under heat stress for both years. Heritability for this trait was noted to be 90.1% and 97.3% during the first year and second year, respectively (**Table 1**).

#### 3.1.5 Leaf area

Significant genetic variation (P<0.01) was noted in leaf area (LA) among the tested genotypes under hot environment for both years (**Supplementary Figure S6**, **Supplementary Table S3**). LA decreased by 23 and 28% in heat-stressed plants, over controls, in the 1^st^ and 2^nd^ years, respectively. Substantial genetic variability for this trait was noted under heat stress environment ranging from 13.9-21.4 cm^2^ (during the first year) and 14.7-20.6 cm^2^ (during second year). High heritability (83.7%) recorded during the first year and 89.8% during second year, suggested that this trait could be used for screening heat tolerance in urdbean (**Table 1**).

### 3.2 Yield and yield-related traits

**Figure 1:**
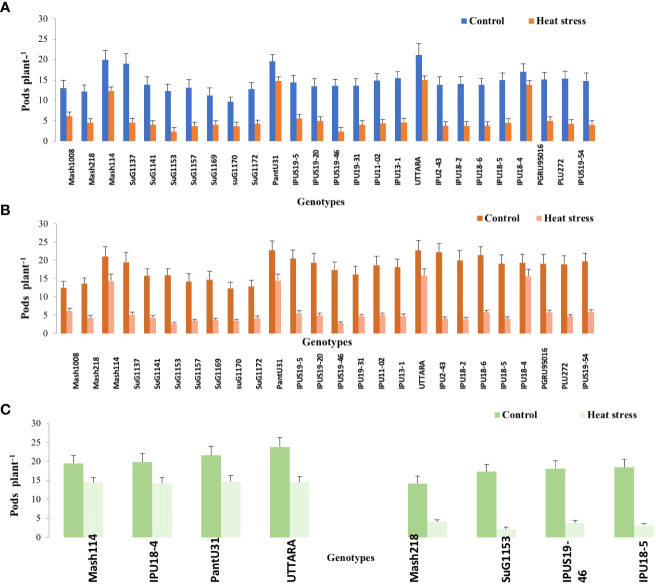
Pod number plant ^-1^ of Urdbean genotypes under control (normal-sown; Control) and heat stress environment during 2018 **(A)**,2019 **(B)** and in controlled environment of growth chamber (GC; **C**). LSD values (P < 0.05); genotype × treatment: 2.6 (2018), 3.1 (2019), 3.46 (GC). Values are means + SE. (n = 3).

**Figure 2:**
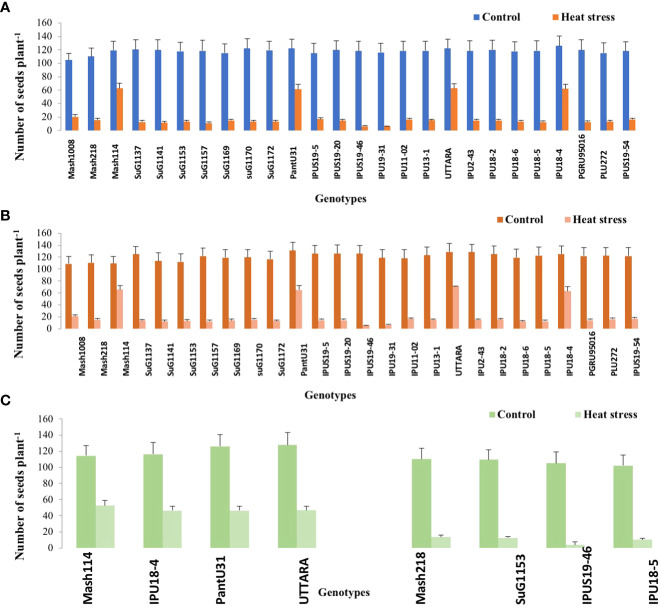
Seed number plant ^-1^ of Urdbean genotypes under control (normal-sown; Control) and heat stress environment during 2018 **(A)**,2019 **(B)** and in controlled environment of growth chamber (**C**; GC). LSD values (P < 0.05); genotype × treatment:6.9 (2018), 7.5 (2019), 12.1 (GC). Values are means + SE. (n = 3).

**Figure 3:**
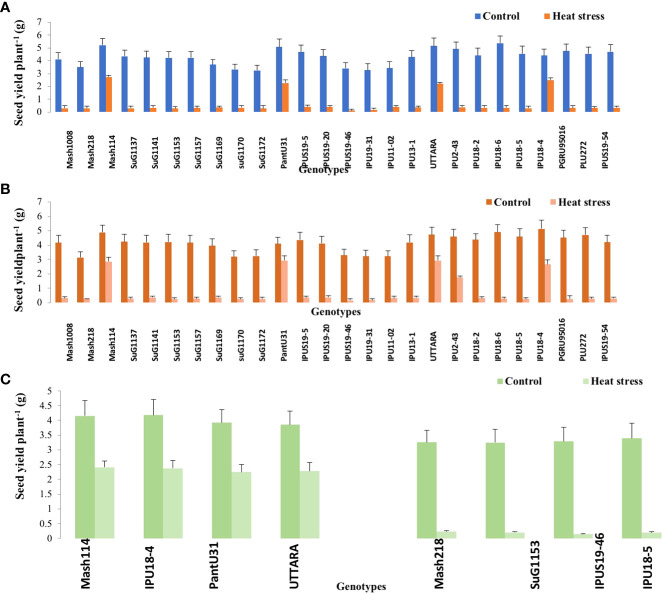
Seed yield plant ^-1^ of Urdbean genotypes under control (normal-sown; Control) and heat stress environment during 2018 **(A)**,2019 **(B)** and in controlled environment of growth chamber (**C**; GC). LSD values (P < 0.05); genotype × treatment: 1.3 (2018), 1.5 (2019), 1.3 (GC). Values are means + SE. (n = 3).

**Figure 4:**
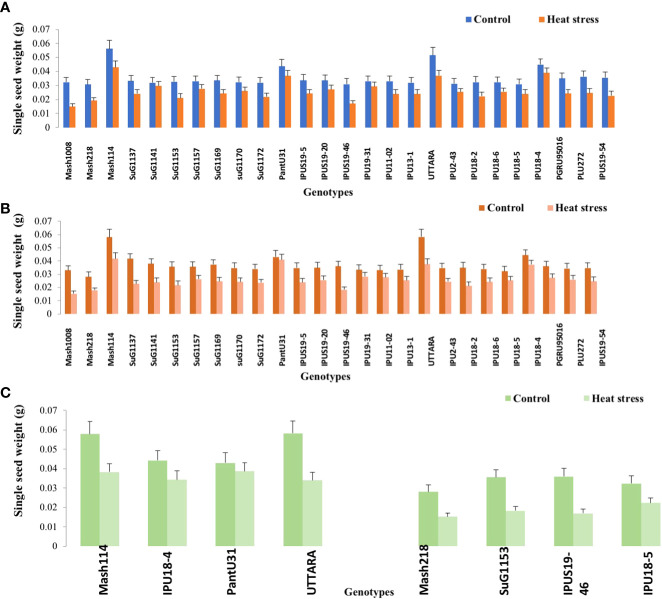
Single seed weight of Urdbean genotypes under control (normal-sown; Control) and heat stress environment during 2018 **(A)**, 2019 **(B)** and in controlled environment of growth chamber (**C**;GC). LSD values (P < 0.05); genotype × treatment: 0.0023 (2018), 0.0021 (2019), 0.0018 (GC). Values are means + SE. (n = 3).

Significant (P<0.01) genetic variability for pods plant^-1^ (**Figure 1**) seeds plant^-1^ (**Figure 2**), seed yield plant^-1^ (**Figure 3**) and single seed weight (**Figure 4**) were recorded under heat stress environment for both years (**Supplementary Table S3**).

Under high temperature, maximum pods plant^-1^ decreased by 82.4 and 83.7%, maximum seeds plant^-1^ by 94.7 and 94.3%, maximum seed yield plant^-1^ by 91.5 and 95.2% and single seed weight by 26 and 32% over the respective controls in 1^st^ and 2^nd^ year, respectively. The UTTARA genotype retained highest pod number plant^-1^ (15, 16) followed by PantU31(15,15), IPU18-04 (14,16) under heat stress environment for both years. For seeds plant^-1^ trait, Mash114 (63, 66), UTTARA (63, 71) and IPU18-04 (62, 63) showed promising results under heat stress environment for both years. Likewise, Mash114 (47.7%, 41.6%), IPU18-04 (43.34%, 48.9%), UTTARA (57.39%, 38%), and PantU31(55.45%, 28.7%) showed lower reduction percentage for seed yield plant^-1^ for both years, and thus could be highly heat tolerant genotypes. Under heat stress environment, high heritability with 98.5%, 99.4% and 99.2% was noted for pods plant^-1^, seeds plant^-1^ and seed yield plant^-1^, respectively during the first year. Similarly, these traits showed high heritability under hot environment during second year also (**Table 1**).

### 3.3 Validation of selected heat tolerant and heat-sensitive Urdbean genotypes in growth chamber

Significant genetic variability for the various evaluated traits was recorded in twenty-six selected urdbean genotypes under normal and heat stress condition in both years (**Supplementary Table S3**). Based on the various physiological and yield and yield related parameters, the following genotypes Mash 114, PantU31, IPU18-04 and UTTARA were identified to be heat tolerant for both years under hot environment. Contrastingly, Mash 118, SuG1153, IPU18-5, IPU5(19-46) were identified to be highly sensitive to heat stress for both years.

To validate the response of selected heat tolerant and sensitive genotypes, a selected set of 4 heat tolerant and 4 heat-sensitive urdbean genotypes among the 26 genotypes were examined under growth chamber subjecting them to normal and heat stress treatments, separately. Among these selected 4 heat tolerant genotypes, Mash114 and IPU18-04 revealed high tolerance to heat stress, evidenced by high number of pods plant^-1^ (14.5,14.2), high seed number plant^-1^ (52.5, 46.5) and high efficiency of various physiological traits (chlorophyll content (**Supplementary Figure S4**), chlorophyll fluorescence (**Supplementary Figure S5**), stomatal conductance (**Supplementary Figure S6**), low electrolyte leakage (**Supplementary Figure S2**), and low malondialdehyde content (**Supplementary Figure S7**) and reproductive traits [high pollen germination (57.4%, 52.5%) and viability percentage (61.2%, 57.1%)] under heat stress environment (see **Supplementary Figure S7**).

However, among the heat-sensitive genotypes, IPU5-(19-46) and IPU-18-5 showed high heat stress sensitivity, evidenced by high reduction of yield and yield-related traits as well as physiological and reproductive traits.

### 3.4 Correlation analysis

Pod number plant^-1^ showed significant positive correlations with traits-chlorophyll content, chlorophyll fluorescence, leaf area and stomatal conductance-directly contributing to photosynthesis process whereas significant and negative association of electrolyte leakage (EL) percentage was noticed (**Table 2**). EL also showed negative correlation with traits (chlorophyll content, chlorophyll fluorescence, leaf area and stomatal conductance), yield traits such as pods plant^-1^, seeds plant^-1^, single seed weight and seed yield plant^-1^ under heat stress environment during both the years.

**Table 2:** Correlation coefficients of various traits with yield traits in plants under heat stress environment.

Trait	Outdoor environment (2018)		Outdoor environment (2019)		Growth Chamber	
	Number of Pods Plant^-1^	Seed yield plant^-1^	Number of Pods Plant^-1^	Seed yield plant^-1^	Number of Pods Plant^-1^	Seed yield plant^-1^
Electrolyte leakage %	-0.71**	-0.76**	-0.73**	-0.73**	-0.96**	-0.98**
Chlorophyll	0.71**	0.72**	0.80**	0.73**	0.98**	0.99**
Chlorophyll fluorescence	0.68**	0.70**	0.87**	0.82**	0.99**	0.99**
Stomatal Conductance	0.91**	0.95**	0.97**	0.95**	0.98**	0.98**
Leaf area	0.70**	0.67**	0.60**	0.54**	0.98**	0.99**
Malondialdehyde					-0.98**	-0.98**
Pollen viability					0.98**	0.97**
Pollen germination					0.96**	0.99**
Number of Pods Plant^-1^	1	0.96**	1	0.95**	1	0.99**
Seed yield plant^-1^	0.96**	1	0.92**	1	0.99**	1
Seeds plant^-1^	0.98**	0.99**	0.98**	0.95**	0.98**	0.99**
Single seed weight	0.81**	0.88**	0.86**	0.85**	0.98**	0.97**

** denotes significant at 1%.

In urdbean plants grown under growth chamber condition, subjected to heat stress, electrolyte leakage and malondialdehyde (an indicator of oxidative stress) showed highly significant negative correlation with all the physiological traits viz., chlorophyll content, chlorophyll fluorescence, stomatal conductance, relative water content, leaf area, pollen germination % and pollen viability % and yield traits viz. pods plant^-1^, seed yield plant^-1^ (**Table 2**).

High and significant positive correlation of pollen germination % and pollen viability % were noticed with all the traits except electrolyte leakage and malondialdehyde. Likewise, stomatal conductance and RLWC also exhibited high and positive correlation with all the traits except electrolyte leakage and malondialdehyde. The yield and yield related traits viz., pods plant^-1^, seeds plant^-1^, single seed weight and seed yield plant^-1^ showed very strong positive correlation with pollen germination and pollen viability (**Table 2**) suggesting these traits as vital for screening heat tolerant urdbean genotypes.

### 3.5 PCA analysis

**Figure 5:**
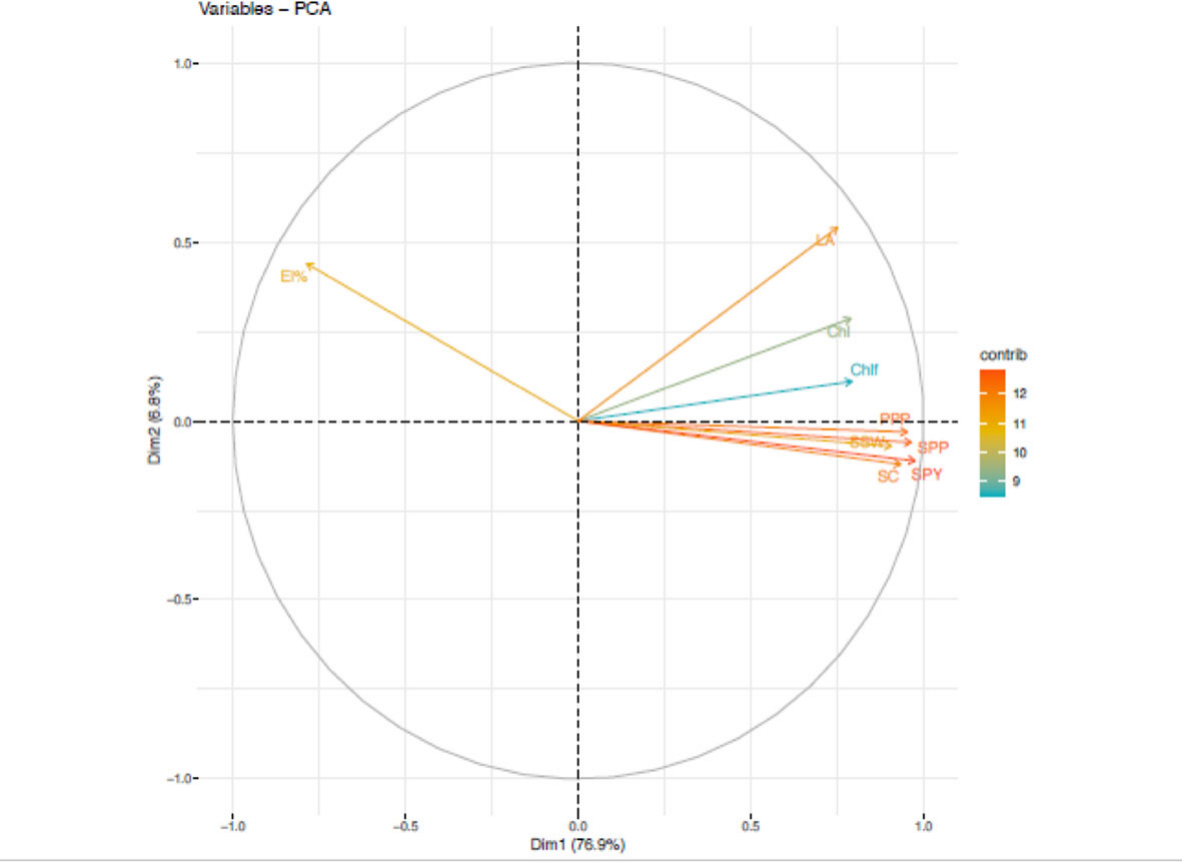
Principal component analysis (PCA) of various traits in Urdbean genotypes under heat stress in the year 2018.

During the first year, under heat stress environment, PCA analysis (**Figure 5**) revealed five principal components correlated to 9 traits accounted for 96.5% of total variability. The individual contribution of each component was 76.8%, 6.84%, 5.18%, 4.23% and 3.38%. Analysis of factor loadings of the traits in the retained PCs suggested that seed yield plant^-1^ (SPY) (13.76), seeds plant^-1^(SPP) (13.45) and pods plant^-1^; PPP (13.12) contributed most positively. In PC2, leaf area (LA) contributed most positively. The trait chlorophyll fluorescence (ChlF) (58.47), chlorophyll (Chl) (76.45) and electrolyte leakage (EL%) (45.1) had highest contribution to PC3, PC4 and PC5, respectively.

**Figure 6:**
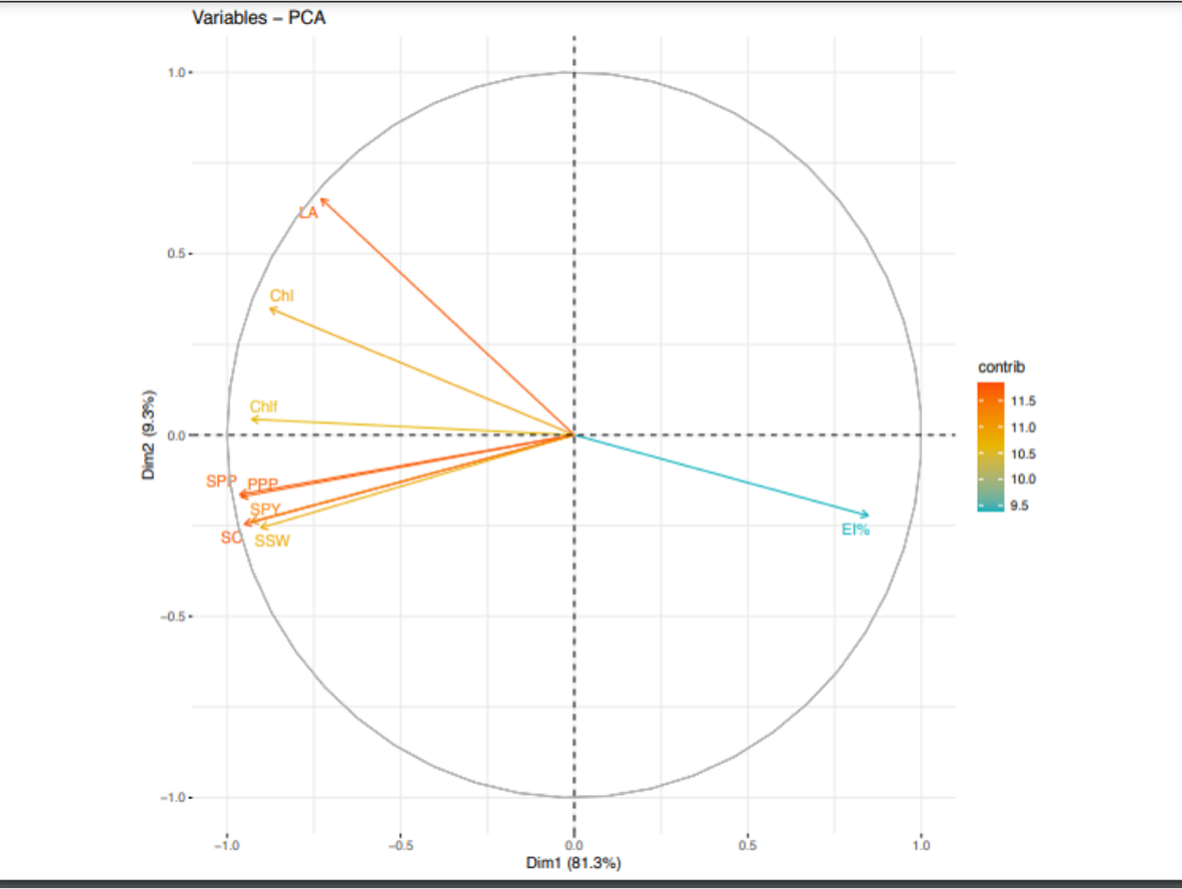
Principal component analysis (PCA) of various traits in urdbean genotypes under heat stress in the year 2019.

Likewise, during second year, PCA analysis (**Figure 6**) indicated five principal components correlating to 9 traits contributed 97.9% to the total variability. The individual contribution of each component was 81.2%, 9.3%, 3.62%, 2.51% and 1.29%. PPP (12.6) had the highest contribution to PC1. Likewise, LA (50.69) contributed with highest positive value to PC2. EL% (57.38), ChlF (40.18) and Chl (53.1) had highest positive contribution to PC3, PC4 and PC5, respectively.

**Figure 7:**
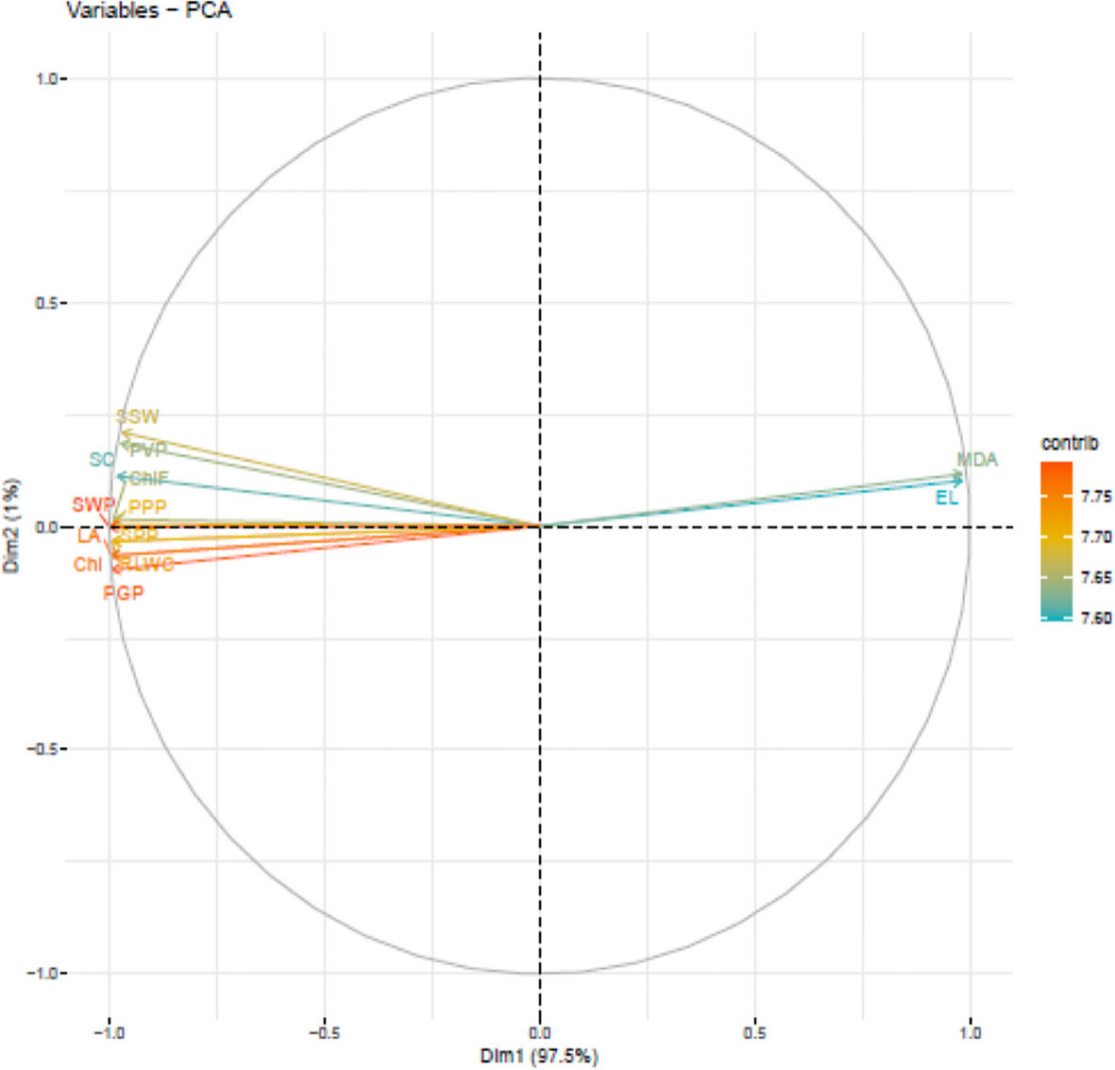
Principal component analysis (PCA) of various traits in urdbean genotypes under heat stress in a growth chamber.

Under growth chamber, PCA analysis (**Figure 7**) suggested five PCAs attributing to 13 traits contributing 99.8% to the total variability. The individual contribution of each component was PC1 (97.5%), PC2 (1.03%), PC3 (0.64%), PC4 (0.35%) and PC5 (0.30%). Chlorophyll content (7.81) had the highest contribution to PC1, while single seed weight (32.58) had the highest contribution to PC2. Electrolyte leakage (30.42%) had the highest contribution to PC3 and stomatal conductance (42.09) had the highest contribution to PC4. Seeds plant^-1^ (32.98) had the highest contribution to PC5.

### 3.6 Clustering and identifying heat tolerant Urdbean genotypes based on outdoor experiments

Based on the heat map analysis considering all the physiological and yield-related traits evaluated during the first year in all, the 26 genotypes revealed three major clusters.

**Figure 8:**
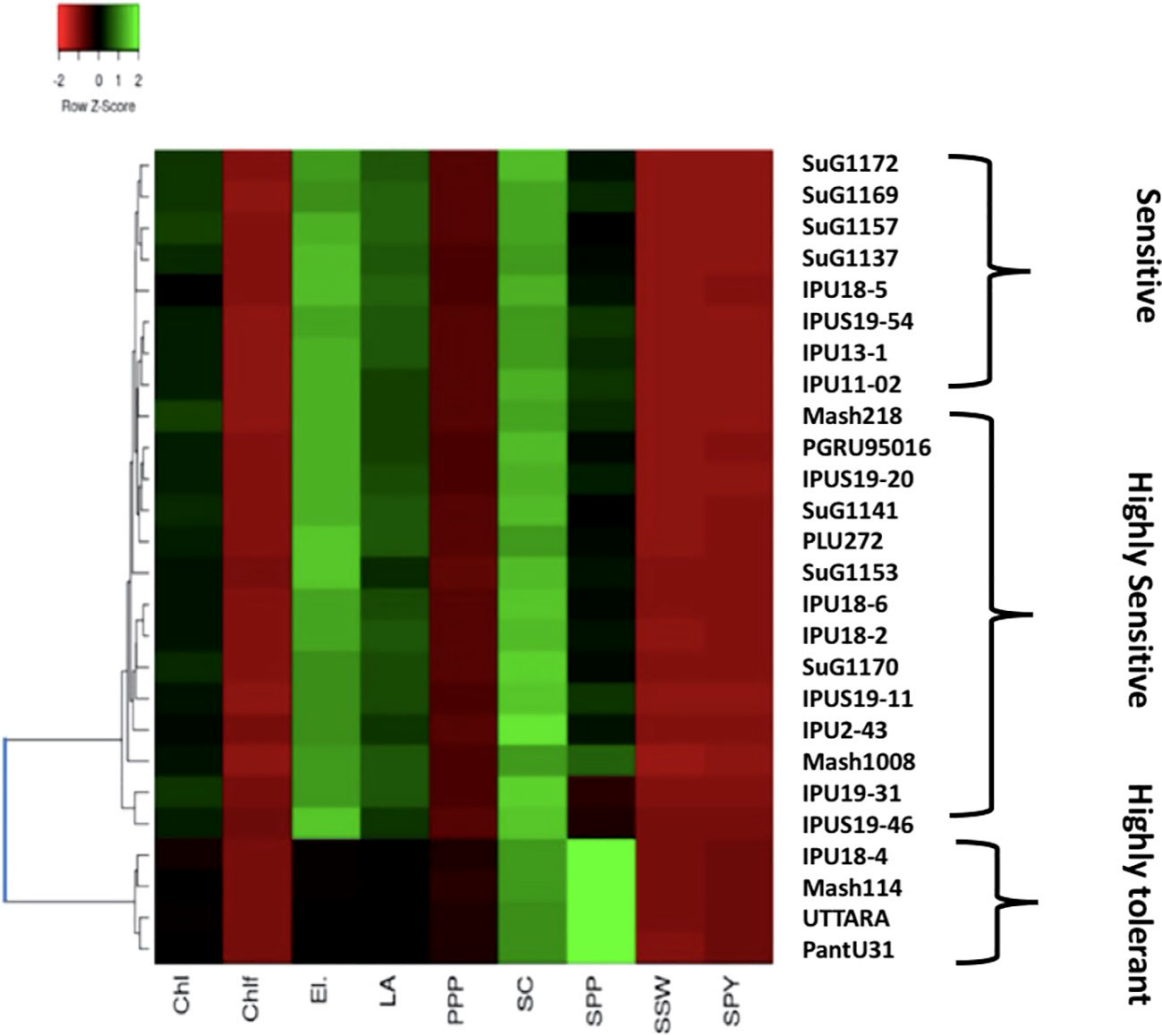
Heat map based on the response of urdbean genotypes to heat stress in the year 2018.

Regarding first year, Cluster 1 contained all the highly heat tolerant genotypes, including IPU18-04(43.34%), Mash 114(47.7%), UTTARA (IPU94-1) (57.39%), and PantU31 (55.45%) (**Figure 8**) relying on low reduction of seed yield plant^-1^ (SYP) compared under non-stress and heat stress conditions. The heat-sensitive genotypes viz., Mash218 (91.35% SYP reduction), IPU5 (96.79% SYP reduction), IPU18-5(93.40% SYP reduction) remained in second cluster. The 3^rd^ cluster had genotypes such as IPU 11-02 (88.45% SYP reduction), SuG1170 (89.79% SYP reduction), SuG1169 (90.235 SYP reduction), Mash 1008 (92.6% SYP reduction)

**Figure 9:**
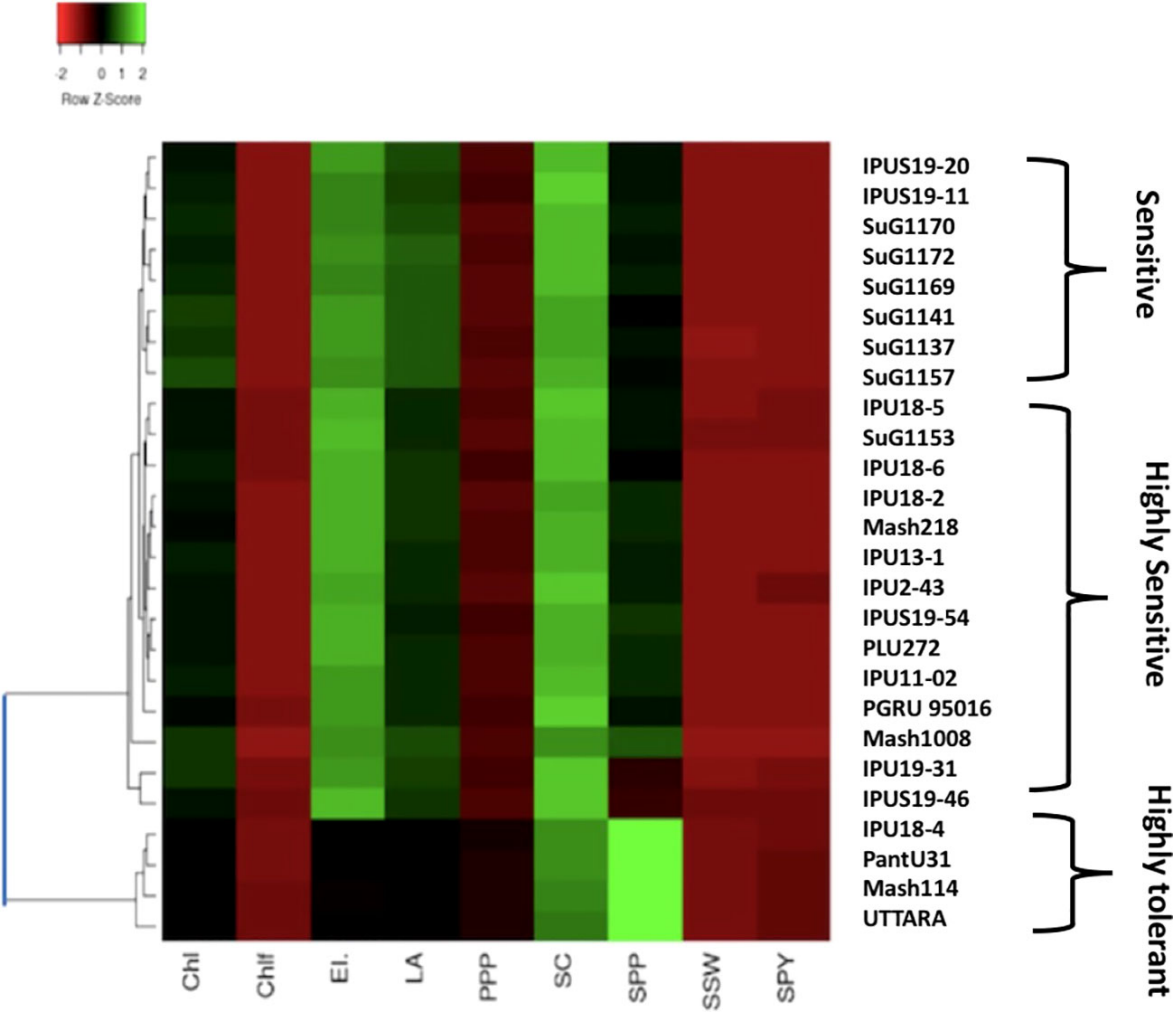
Heat map based on the response of urdbean genotypes to heat stress in the year 2019.

Likewise, during second year, the heat map also divided the genotypes into three clusters (**Figure 9**). The first cluster contained the highly heat tolerant genotypes viz., Mash 114 (41.6% SYP reduction), PantU31 (28.7% SYP reduction), IPU18-04 (48.35% SYP reduction) and UTTARA (38% SYP reduction). In the second cluster, all the heat-sensitive genotypes such as Mash 218 (91.5% SYP reduction), SuG1153 (94.1% SYP reduction), IPU18-5 (94.8% SYP reduction) and IPU5 (95.2% SYP reduction) were placed. Other genotypes, for example, IPU2-43 (62.1% SYP reduction), IPU-11-02 (90.3% SYP reduction), and SuG1169 (90.8% SYP reduction) were found in third cluster.

**Figure 10:**
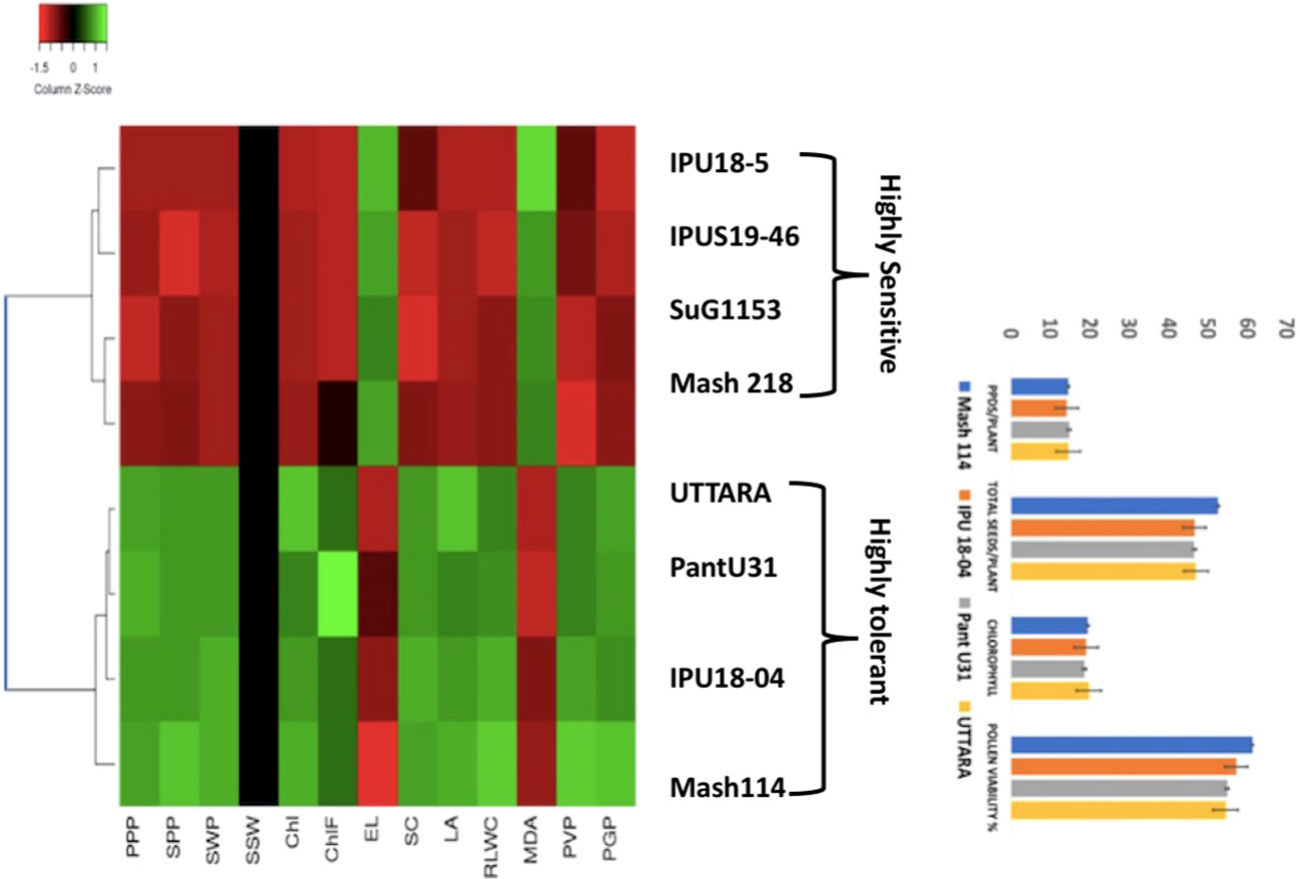
Heat map based on the response of urdbean genotypes to heat stress in a growth chamber.

The cluster analysis of the selected genotypes evaluated for various traits under growth chamber condition resulted in two major clusters (**Figure 10**). The first cluster contained all the four heat-sensitive genotypes such as IPU18-5, IPU5, SuG1153, and Mash 218, whereas the second cluster contained all the heat tolerant genotypes UTTARA, PantU31, IPU18-04, and Mash114.

**Figure 11:**
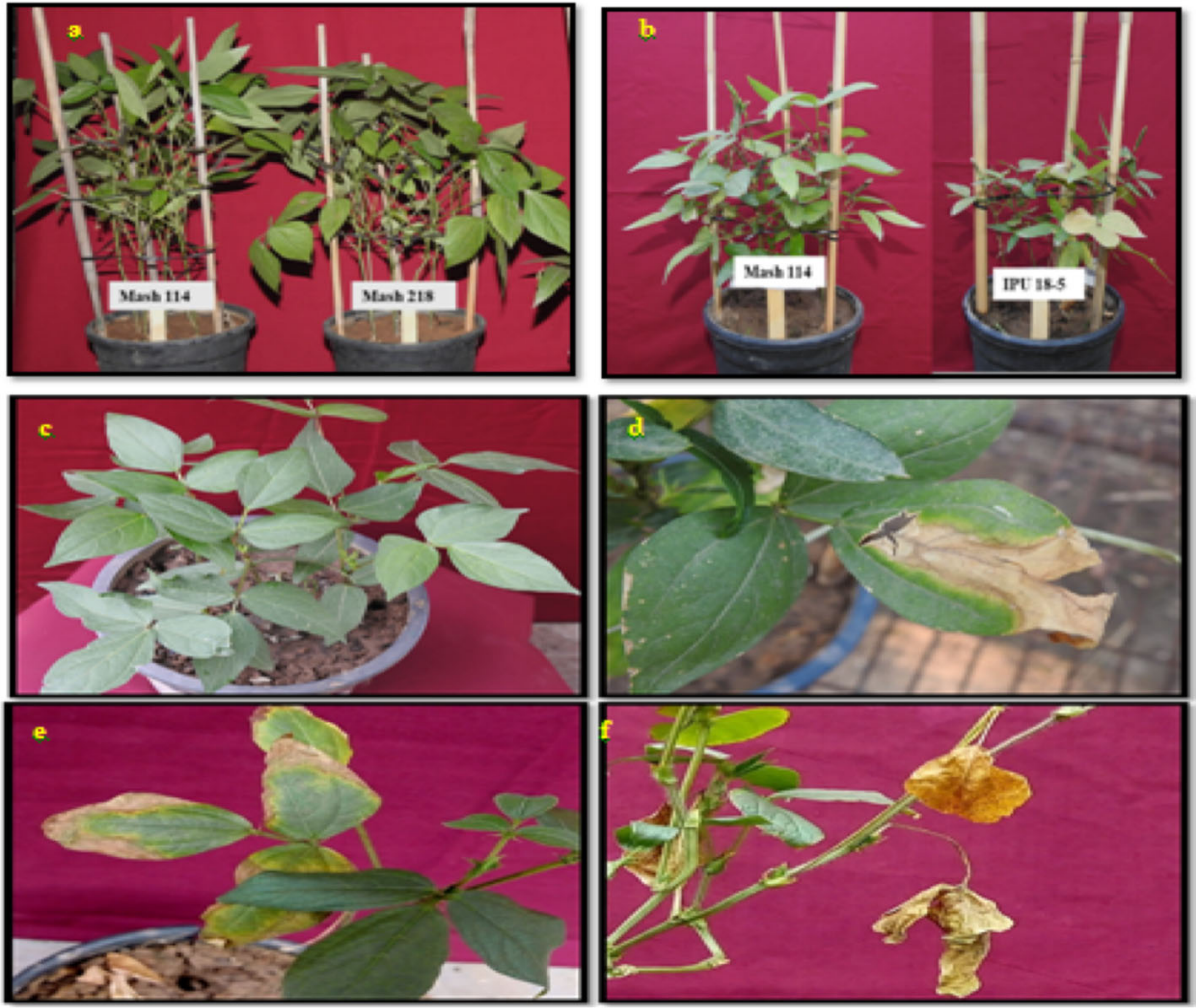
Morphological effects of heat stress on Urdbean plants; plant height under control environment **(A)**, reduced plant height under heat stress (HS) environment **(B)**, healthy leaves under control environment **(C)**, scorching of leaves under HS **(D)**, chlorosis in the HS **(E)**, Leaf senescence and abscissionin the HS **(F)**.

**Figure 12:**
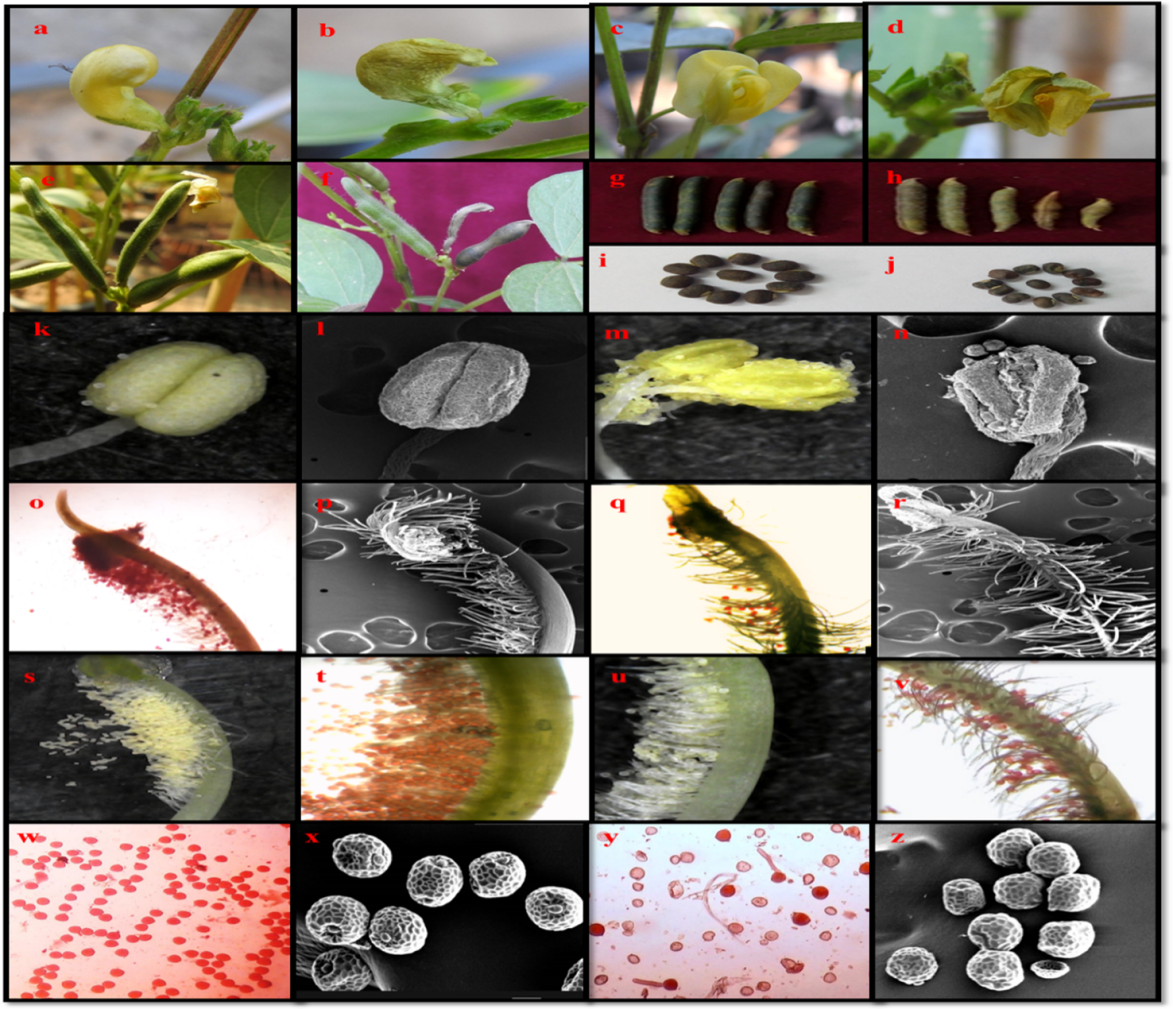
Urdbean plants showing various distinctive impacts on it are the reproductive phase when raised under control and heat stress environment. Plants grown under control temperature have healthy bud **(A)** healthy flower **(C)** filled pods (egg), normal seeds **(I)** compact anther **(K, L)** receptive stigma **(O, P)** higher pollen load **(S, T)** and possess viable pollen grains **(W, X)**. However, plants raised under heat stress conditions have more frequency of aborted buds **(B)** aborted flowers **(D)** unfilled and aborted pods **(F, H)** shrivelled seeds **(J)** distorted anther **(M, N)** non-receptive stigma **(Q, R)** less pollen load **(U, V)** and non-viable pollen grains **(Y, Z)** healthy flower bud **(A)** aborted flower bud **(B)** healthy flower **(C)** aborted flower **(D)** healthy pods **(E)** aborted pod **(F)** normal pod length **(G)** reduced pod length **(H)** healthy seeds **(G)** shrivelled seeds **(H)** healthy anther under stereo-microscope **(I)** healthy anther under SEM **(J)** distorted anther.

Various symptoms of heat stress on urdbean at vegetative and reproductive growth are shown in **Figures 11** and **12**.

## 4 Discussion

Increasing frequency of heat stress events poses serious challenges in all the plant growth stages, especially, reproductive stage, resulting in significant yield loss in various crop plants, including urdbean (Jha et al., 2014; Jha et al., 2017; Chaudhary et al., 2020; Chaudhary et al., 2022). Thus, assessing urdbean’s genetic variability for phenological, morpho-physiological, biochemical and yield and yield related traits is one of the prime objectives for developing heat tolerant climate resilient urdbean genotypes.

A selected set of 26 urdbean genotype were examined for heat stress tolerance by growing them under non-stress and heat stress under field condition and under controlled growth chamber conditions. Wide range of genetic variability for various physiological, biochemical and yield and yield related traits was observed. Thus, some genotypes showing promising results based on the physiological traits such as chlorophyll content, chlorophyll fluorescence, and yield traits like high pod setting plant^-1^ and high seed yield plant^-1^ under heat stress environment were identified and validated for heat tolerance under controlled high temperature environment. These urdbean genotypes could be a precious resource for heat tolerance. Further, the tolerant urdbean genotypes could be potentially used for investigating the genetic control of heat tolerance in urdbean.

Among the various physiological traits for assessing heat stress response at vegetative stage during photosynthesis, measuring chlorophyll content, electrolyte leakage, stomatal conductance and photosystem II function are essential parameters for selecting heat tolerant genotypes (Srinivasan et al., 1996; Sita et al., 2017; Bhandari et al., 2020; Devi et al., 2022). Chlorophyll is the main photosynthetic pigment, assists in capturing light energy and enables in photosynthesis process (Wang et al., 2018). Heat stress causes reduction in Chl content resulting in leaf senescence (Kim and Nam, 2007). Improved retention of Chl content under heat stress could be an indication of heat stress tolerance. Thus, genetic variability for Chl content could be useful for selecting heat tolerant genotypes. Heat tolerance based on membrane stability measured by electrolyte/ion leakage is an important trait for selecting genotypes for heat tolerance (Bajji et al., 2002). Under high temperature stress, the genotypes showing low electrolyte leakage indicate stable cell membrane stability and thus are considered heat tolerant. Likewise, stomatal conductance and transpiration cooling are important traits for assessing heat tolerance in plants. Leaf cooling is a vital heat stress avoidance mechanism (Deva et al., 2020) thus, enhanced stomatal conductance and transpiration cooling could help plants to conduct photosynthesis process under high temperature stress (Porch and Hall, 2013). Thus, genotypes with high stomatal conductance could be heat tolerant. In the current study, Mash114, UTTARA, PantU31 genotypes showed high chlorophyll content, high stomatal conductance, low electrolyte leakage and high Fv/Fm value under heat stress, both under field and growth chamber condition, indicating their heat stress tolerance. Similar findings were reported in chickpea (Devi et al., 2022), lentil (Srinivasan et al.,1996; Delahunty et al., 2015; Sita et al., 2017; Sehgal et al., 2019; Bhandari et al., 2020) and pea (McDonald and Paulsen, 1997) under heat stress.

Of all the growth stages, reproductive stage is the most sensitive stage affected by negative impact of heat stress (Zinn et al., 2010). High temperature stress causes anomalies and malfunction in reproductive processes ranging from reduction in pollen germination (PGP) percentage, pollen viability percentage (PVP), malformation in ovule to inhibition in fertilization process in various crops, including rice (Xu et al., 2021), wheat (Ullah et al., 2022), chickpea (Bhandari et al., 2020; Devi et al., 2022), common bean (Silva et al., 2019; Soltani et al., 2019) and tomato (Gonzalo et al., 2021). High PGP and PVP values are indicators of efficient reproductive function leading to high pod and seed setting resulting in improved yield under heat stress (Firon et al., 2006; Pham et al., 2020). Sufficient range of genetic variability for PGP (15.4%-57.4%), PVP (24.5%-61.2%) was noted under heat stress in the present study, providing scope for selection and developing heat tolerant urdbean genotypes. Based on these traits, Mash114 and PantU31 genotypes could be promisingly used as donor parents for improving heat tolerance in urdbean. Screening of heat tolerance relying on PGP and PVP has been reported in rice (Zhang et al., 2018), wheat (Bheemanahalli et al., 2019), chickpea (Devi et al., 2022), lentil (Barghi et al., 2013), common bean (Silva et al., 2019; Vargas et al., 2021), tomato (Pham et al., 2020) and sorghum (Djanaguiraman et al., 2018).

Emphasizing on yield and yield-related parameters such as pods plant^-1^, significant genetic variability ranging from (2.43-15.07 during the first year) and (2.77-15.9 during second year), for seed yield plant^-1^ (0.11-2.73g during the first year) and (0.16-2.93g during the second year) and for total seeds plant^-1^ (6.2-62.9 during the first year) and (6.7-71.2 during the second year) under heat stress was recorded. Thus, genotypes with high pod setting, high seed yield plant^-1^ and high seed number plant^-1^ under heat stress environment could be promisingly selected as heat tolerant genotypes. Based on these traits, Mash114, PantU31, UTTARA and IPU18-04 were selected as heat tolerant urdbean genotypes. Similarly, in previous studies, based on these yield traits, genotypes “40–10,” “Naparnyk,” and “CDC Meadow” in pea (Jiang et al., 2020), G122, PI 163120, Cornell 503 in common bean (Shonnard and Gepts, 1994; Rainey and Griffiths, 2005a), ICC1205, ICC15614, GNG469, GNG1488, GNG1499, and GNG1969 in chickpea (Devasirvatham et al., 2013), B89-200 and TN88-63 in cowpea (Ehlers and Hall, 1998), 72578, 70548, 71457 and 73838 in lentil (Delahunty et al.,2015) and 55–437 and 796 in groundnut (Ntare et al., 2001) were identified to be heat tolerant.

Correlation studies indicated that electrolyte leakage trait had highly negative association with all the physiological (except MDA) and yield and yield-related traits under heat stress condition, indicating genotypes having high value for electrolyte leakage are highly heat- sensitive genotypes. However, other physiological traits viz., chlorophyll content, chlorophyll florescence, relative water content stomatal conductance showing high and positive association with yield and yield related traits viz., seed yield plant^-1^, total seeds plant^-1^ and single seed weight indicated that selection of urdbean genotypes with high chlorophyll content, enhanced stomatal conductance and high relative water content under heat stress could be highly heat tolerant. Positive association of chlorophyll content, stomatal conductance trait related to photosynthesis process with yield and yield-related traits ranging from seed yield plant^-1^, pod number plant^-1^ and single seed weight under heat stress has been reported in chickpea (Devi et al., 2022), lentil (Sita et al., 2017), and common bean (Petkova et al., 2007).

Studies conducted in controlled high temperature environment of growth chamber revealed PGP and PVP to be highly correlated with pods plant^-1^ and could be used as vital indicators of heat tolerance. Earlier studies have also indicated that these traits could be used for selecting heat tolerant genotypes in chickpea (Devi et al., 2022), common bean (Rainey and Griffiths, 2005), lentil (Sehgal et al., 2019) and tomato (Gonzalo et al., 2021).

High heritability of various morpho-physiological, yield and yield related traits could be of great importance for selecting these traits for screening of heat tolerant genotypes in various crops. High heritability for chlorophyll content, stomatal conductance, seed yield plant^-1^, pods plant^-1^, single seed weight, as noticed in the present study has also been noted in heat tolerant chickpea (Jha et al., 2019; Devi et al., 2022), rice (Enzi et al., 2022), tomato (Panthee et al., 2018), wheat (Rebetzke et al., 2013) under high temperature environment.

## 5 Conclusion

Heat stress related events are becoming serious constraints for crop yield including urdbean thus, causing great concern for global food security. Harnessing the genetic variability for various morpho-physiological and yield and yield related traits existing across the crop gene pool could be one of the important approaches for developing heat tolerant crop cultivars including urdbean. A wide range of genetic variability for various morpho-physiological and yield and yield related traits were captured for a selected 26 urdbean genotypes under both non-stress and heat stress environment for consecutive two years. A selected four heat tolerant and four heat-sensitive genotypes were further validated for their heat stress response under controlled growth chamber condition. Based on the results obtained from both outdoor and controlled growth chamber conditions, yield and yield related traits viz., pods plant^-1^, seeds plant^-1^, single seed weight and seed yield plant^-1^ showed strong positive correlation with chlorophyll, chlorophyll fluorescence, and stomatal conductance. Similarly, these yield traits had very strong correlation with reproductive traits, pollen germination and pollen viability except electrolyte leakage and malondialdehyde content. These results indicated selection for high pollen germination % and high pollen viability % and yield and yield related traits could assist in selecting heat tolerant urdbean genotypes. Thus, the candidate genotypes PantU31, Mash114, UTTARA and IPU18-04 exhibiting high pod setting and high seed yield plant^-1^ under heat stress imposed under outdoor and growth chamber environment could be potentially used as heat tolerant donor parents for future urdbean breeding programme. Further, these genotypes can be assessed for their heat tolerance across the multiple locations for confirming their heat tolerance based on various locations.

## Data availability statement

The original contributions presented in the study are included in the article/**Supplementary Material**. Further inquiries can be directed to the corresponding authors.

## Author contributions

SC conducted the experiment. UJ and PJP helped in analysis and writing part of the manuscript. SS and DS contributed in providing the urdbean lines. KS and SK assisted in conducting the biochemical analyses. HN conceived the idea and helped in writing the manuscript. KHMS helped in writing part of the manuscript and edited the manuscript. All authors contributed to the article and approved the submitted version.

## Acknowledgments

SC thanks CSIR for project fellowship and CSIR-UGC for providing a doctoral research fellowship during her course study. The corresponding author HN is thankful to DST (FIST and PURSE), UGC, DBT, CSIR, India, The University of Western Australia (Australia), IIPR (Kanpur, India), PAU (Ludhiana, India), for supporting the research work at various times.

## Conflict of interest

The authors declare that the research was conducted in the absence of any commercial or financial relationships that could be construed as a potential conflict of interest.

## Publisher’s note

All claims expressed in this article are solely those of the authors and do not necessarily represent those of their affiliated organizations, or those of the publisher, the editors and the reviewers. Any product that may be evaluated in this article, or claim that may be made by its manufacturer, is not guaranteed or endorsed by the publisher.
