# Supplementary material for: Assessing the heat sensitivity of Urdbean (Vigna mungo L. Hepper) genotypes involving physiological, reproductive and yield traits under field and controlled environment
Source: Front Plant Sci. 2022 Nov 21;13:1042999. doi: 10.3389/fpls.2022.1042999 (PMC9733429; doi:10.3389/fpls.2022.1042999)
Supplement: Supplementary file 1 [file DataSheet_1.pdf]

Supplementary Table S1: List of Urdbean genotypes used in the study

Sr. No.	Genotypes	Source
**1**	**Mash 1008**	Punjab Agricultural University, Ludhiana, India
**2**	**Mash 218**	Punjab Agricultural University, Ludhiana, India
**3**	**Mash 114**	Punjab Agricultural University, Ludhiana, India
**4**	**SuG 1137**	Punjab Agricultural University, Ludhiana, India
**5**	**SuG 1141**	Punjab Agricultural University, Ludhiana, India
**6**	**SuG 1153**	Punjab Agricultural University, Ludhiana, India
**7**	**SuG 1157**	Punjab Agricultural University, Ludhiana, India
**8**	**SuG 1169**	Punjab Agricultural University, Ludhiana, India
**9**	**SuG 1170**	Punjab Agricultural University, Ludhiana, India
**10**	**SuG 1172**	Punjab Agricultural University, Ludhiana, India
**11**	**Pant U31**	Punjab Agricultural University, Ludhiana, India
**12**	**IPUS19-11**	Indian Institute of Pulses Research, Kanpur, India
**13**	**IPUS19-20**	Indian Institute of Pulses Research, Kanpur, India
**14**	**IPUS19-46**	Indian Institute of Pulses Research, Kanpur, India
**15**	**IPU 19-31**	Indian Institute of Pulses Research, Kanpur, India
**16**	**IPU 11-02**	Indian Institute of Pulses Research, Kanpur, India
**17**	**IPU 13-1**	Indian Institute of Pulses Research, Kanpur, India
**18**	**UTTARA (IPU 94-1)**	Indian Institute of Pulses Research, Kanpur, India
**19**	**IPU 2-43**	Indian Institute of Pulses Research, Kanpur, India
**20**	**IPU 18-2**	Indian Institute of Pulses Research, Kanpur, India
**21**	**IPU 18-6**	Indian Institute of Pulses Research, Kanpur, India
**22**	**IPU 18-5**	Indian Institute of Pulses Research, Kanpur, India
**23**	**IPU 18-04**	Indian Institute of Pulses Research, Kanpur, India
**24**	**PGRU- 95016**	Indian Institute of Pulses Research, Kanpur, India
**25**	**PLU-272**	Indian Institute of Pulses Research, Kanpur, India
**26**	**IPUS19-54**	Indian Institute of Pulses Research, Kanpur, India

Supplementary Table S2: Maximum, minimum temperature, relative humidity (RH) and light intensity during normal and late-sown (heat-stressed) the years 2018 and 2019

	**Season**	**Growth stage**	**Average Max/Min Temp RH**
1.	2018 Normal Sown	Reproductive stage	27.6.4-31.4°C (maximum) and 19.4-25.6°C (minimum)<br>56.7-74.6% (maximum) and 24.5-57.8% (minimum)
2.	2018 Late-sown (heat-stressed)<br>Light intensity: 1,321–1,469 µmol m^{-2} s^{-1}	Reproductive stage	35.4-45.4°C (maximum) and 32.9-36.9°C (minimum)<br>41.3-61.3% (maximum) and 22.5-46.7.1% (minimum)
3.	2019 Normal Sown	Reproductive stage	18.3-32.4°C (maximum) and 9.2-17.3°C (minimum)<br>73-84% (maximum) and 17-60% (minimum)
4.	2019 Late-Sown (heat-stressed)	Reproductive stage	37.3-.42.6°C (maximum) and 33.3-37.5°C (minimum)<br>38.6-57.4% (maximum) and 21.5-42.3% (minimum)

Light intensity: 1,451–1,562 µmol m^{-2} s^{-1}

Supplementary Table S3
ANOVA for various traits recorded in urd bean genotypes grown outdoors in 2018 and 2019, and in a growth chamber under controlled conditions.

	Outdoor environment 2018			Outdoor environment 2019			Growth chamber		
	**T**	**G**	**Tx G**	**T**	**G**	**TxG**	**T**	**G**	**TxG**
Chlorophyll content	<0.01	<0.01	<0.01	<0.01	<0.01	<0.01	<0.01	<0.01	<0.01
Chlorophyll fluorescence	<0.01	<0.01	<0.01	<0.01	<0.01	<0.01	<0.01	<0.01	<0.01
Electrolyte leakage%	<0.01	<0.01	<0.01	<0.01	<0.01	<0.01	<0.01	<0.01	<0.01
Leaf area	<0.01	<0.01	<0.01	<0.01	<0.01	<0.01	<0.01	<0.01	<0.01
Pods plant^{-1}	<0.01	<0.01	<0.01	<0.01	<0.01	<0.01	<0.01	<0.01	<0.01
Stomatal conductance	<0.01	<0.01	<0.01	<0.01	<0.01	<0.01	<0.01	<0.01	<0.01
Seeds plant^{-1}	<0.01	<0.01	<0.01	<0.01	<0.01	<0.01	<0.01	<0.01	<0.01
Seed yield plant^{-1}	<0.01	<0.01	<0.01	<0.01	<0.01	<0.01	<0.01	<0.01	<0.01
Single seed weight	<0.01	<0.01	<0.01	<0.01	<0.01	<0.01	<0.01	<0.01	<0.01

significance level
$p<0.01$

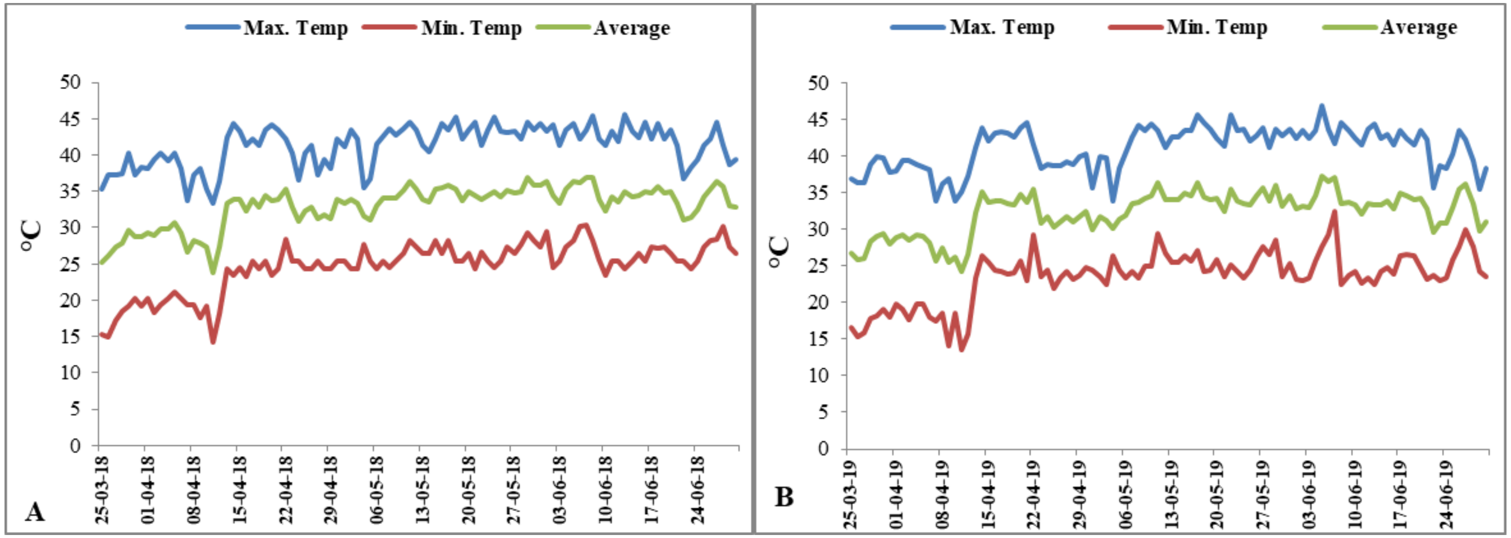

Max. Temp
Min. Temp
Average
°C
A
B

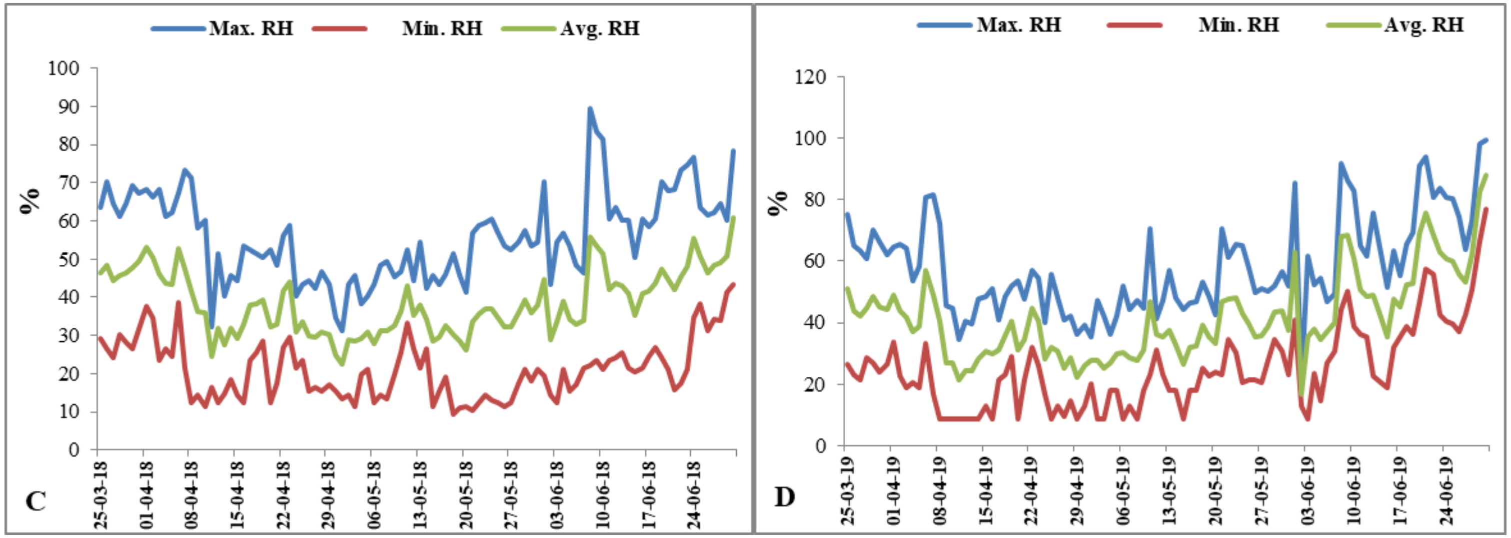

Supplementary Fig.S1: Weather data (maximum (Max), minimum (Min) and average (Avg) temperature (°C), [A (2018, B(2019)] and relative humidity RH (%) [(C(2018), D (2019].

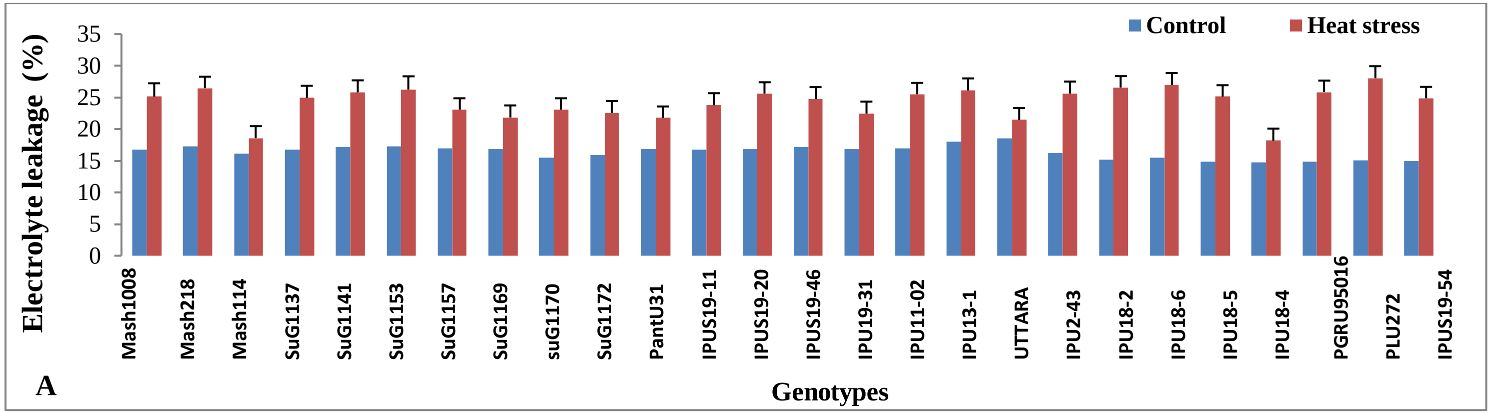
Control
Heat stress
Electrolyte leakage (%)
35
30
25
20
15
10
5
0
Mash1008
Mash218
Mash114
SuG1137
SuG1141
SuG1153
SuG1157
SuG1169
suG1170
SuG1172
PantU31
IPUS19-11
IPUS19-20
IPUS19-46
IPU19-31
IPU11-02
IPU13-1
UTTARA
IPU2-43
IPU18-2
IPU18-6
IPU18-5
IPU18-4
PGRU95016
PLU272
IPUS19-54
A
Genotypes

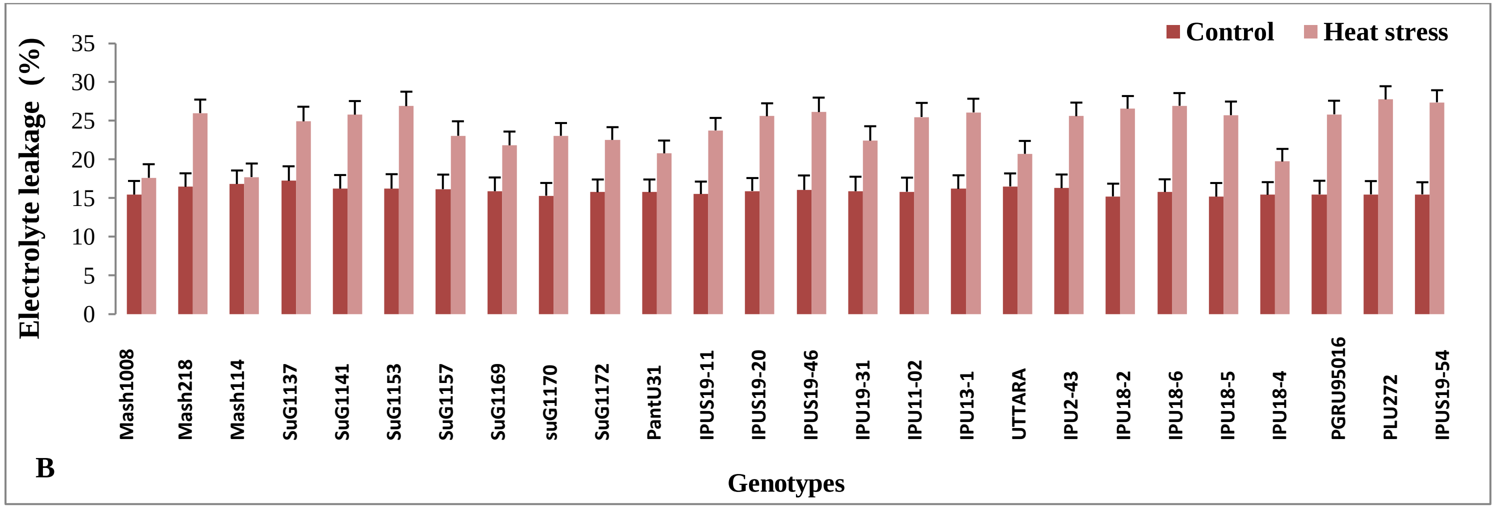
Control
Heat stress
Electrolyte leakage (%)
35
30
25
20
15
10
5
0
Mash1008
Mash218
Mash114
SuG1137
SuG1141
SuG1153
SuG1157
SuG1169
suG1170
SuG1172
PantU31
IPUS19-11
IPUS19-20
IPUS19-46
IPU19-31
IPU11-02
IPU13-1
UTTARA
IPU2-43
IPU18-2
IPU18-6
IPU18-5
IPU18-4
PGRU95016
PLU272
IPUS19-54
B
Genotypes

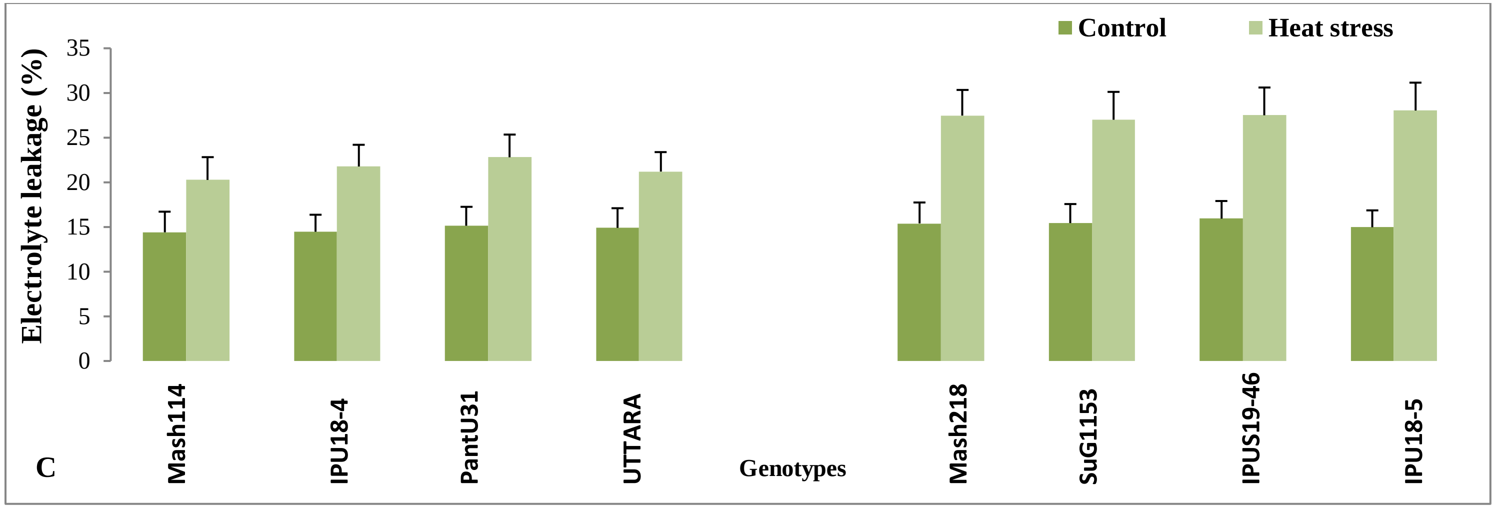

Supplementary Fig.S2: Electrolyte leakage of Urdbean genotypes under control (normal-sown; Control) and heat stress environment during 2018 (A),2019 (B) and in controlled environment of growth chamber (C;GC). LSD values ($P < 0.05$); genotype × treatment: 3.5 (2018), 2.9 (2019), 3.13 (GC). Values are means + SE. (n = 3).

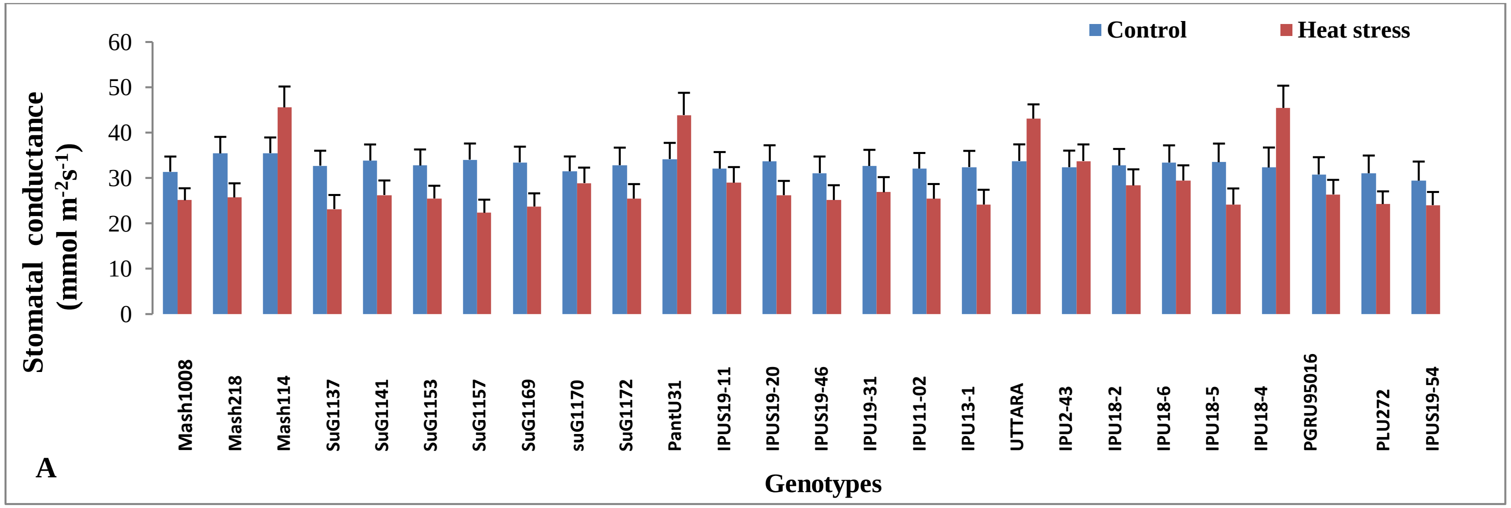

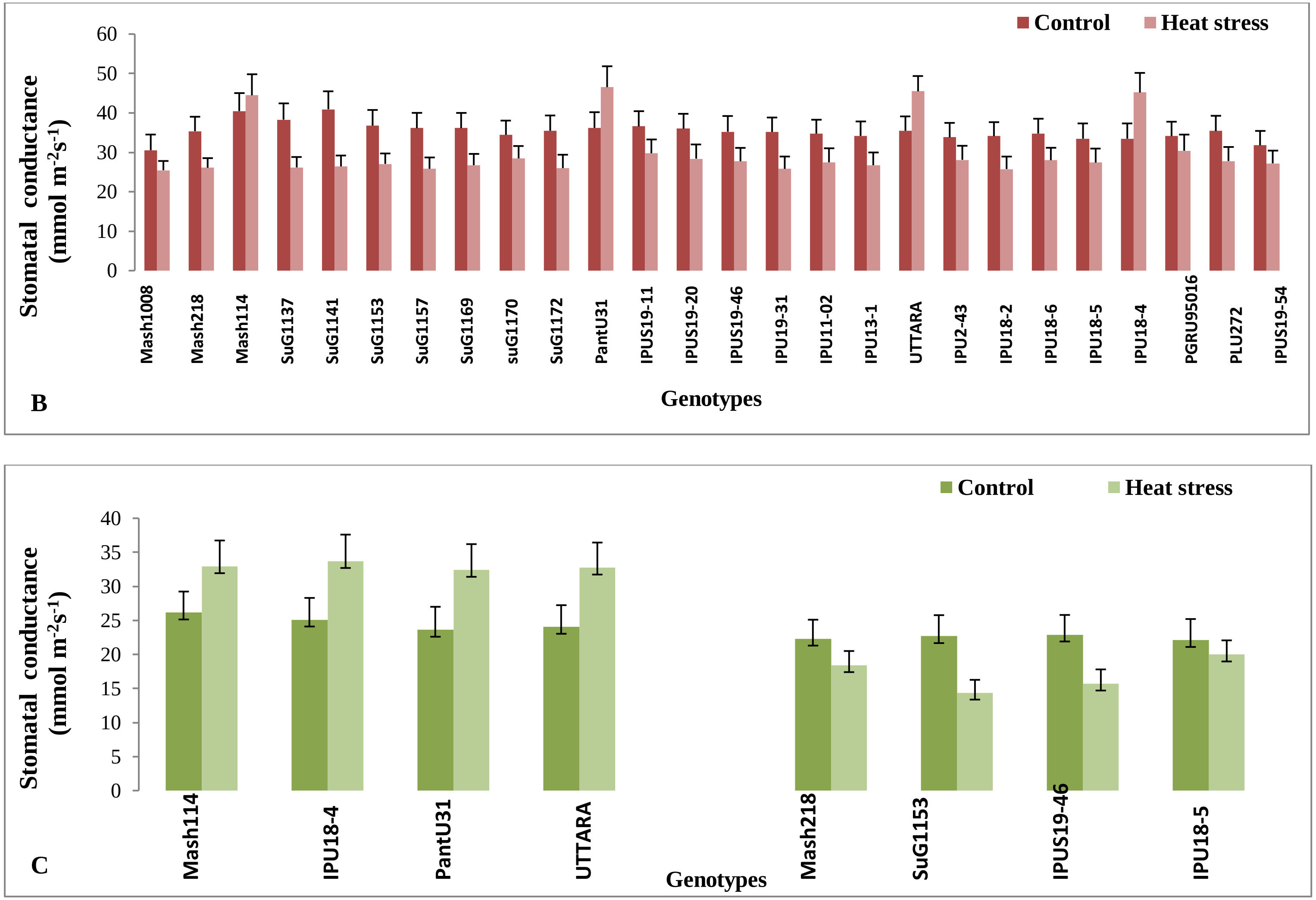

Supplementary Fig.S3: Leaf Stomatal conductance of Urdbean genotypes under control (normal-sown; Control) and heat stress environment during 2018 (A),2019 (B) and in controlled environment of growth chamber (C; GC). LSD values ($P < 0.05$); genotype × treatment: 3.9 (2018), 3.1 (2019), 2.14 (GC). Values are means + SE. (n = 3).

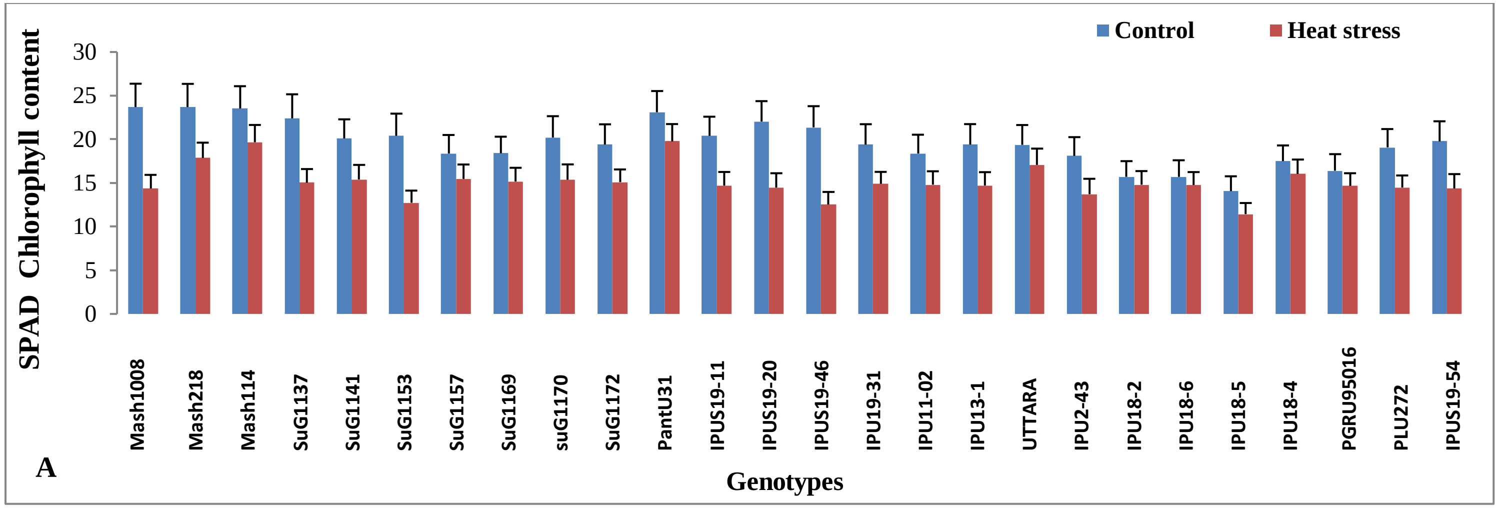

Control
Heat stress
SPAD Chlorophyll content
30
25
20
15
10
5
0
Mash1008
Mash218
Mash114
SuG1137
SuG1141
SuG1153
SuG1157
SuG1169
suG1170
SuG1172
PantU31
IPUS19-11
IPUS19-20
IPUS19-46
IPU19-31
IPU11-02
IPU13-1
UTTARA
IPU2-43
IPU18-2
IPU18-6
IPU18-5
IPU18-4
PGRU95016
PLU272
IPUS19-54
A
Genotypes

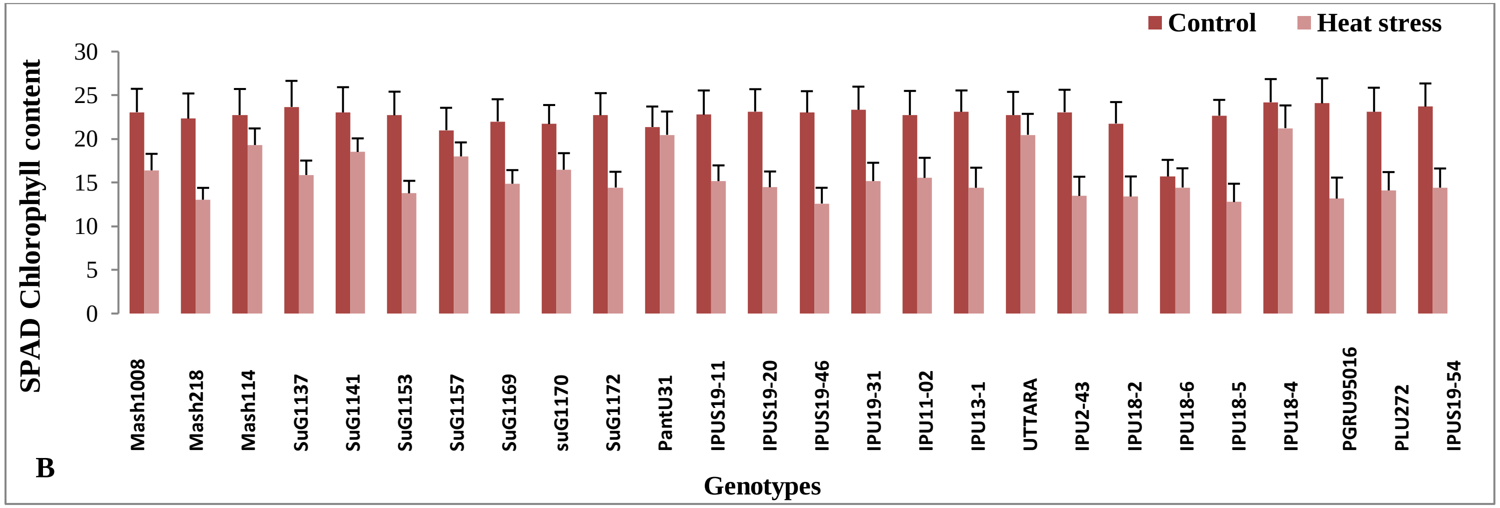

Control
Heat stress
SPAD Chlorophyll content
30
25
20
15
10
5
0
Mash1008
Mash218
Mash114
SuG1137
SuG1141
SuG1153
SuG1157
SuG1169
suG1170
SuG1172
PantU31
IPUS19-11
IPUS19-20
IPUS19-46
IPU19-31
IPU11-02
IPU13-1
UTTARA
IPU2-43
IPU18-2
IPU18-6
IPU18-5
IPU18-4
PGRU95016
PLU272
IPUS19-54
B
Genotypes

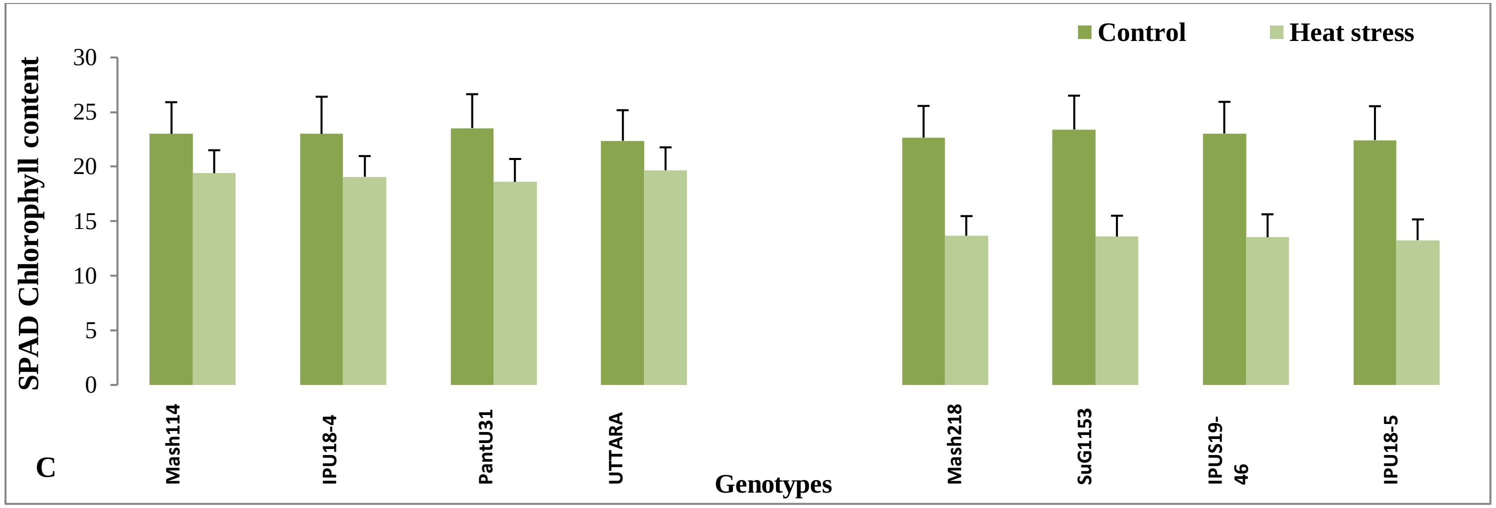

Supplementary Fig.S4: SPAD chlorophyll of Urdbean genotypes under control (normal-sown; Control) and heat stress environment during 2018 (A),2019 (B) and in controlled environment of growth chamber (C;GC). LSD values ($P < 0.05$); genotype × treatment: 2.76 (2018), 2.15 (2019), 1.4 (GC). Values are means + SE. (n = 3).

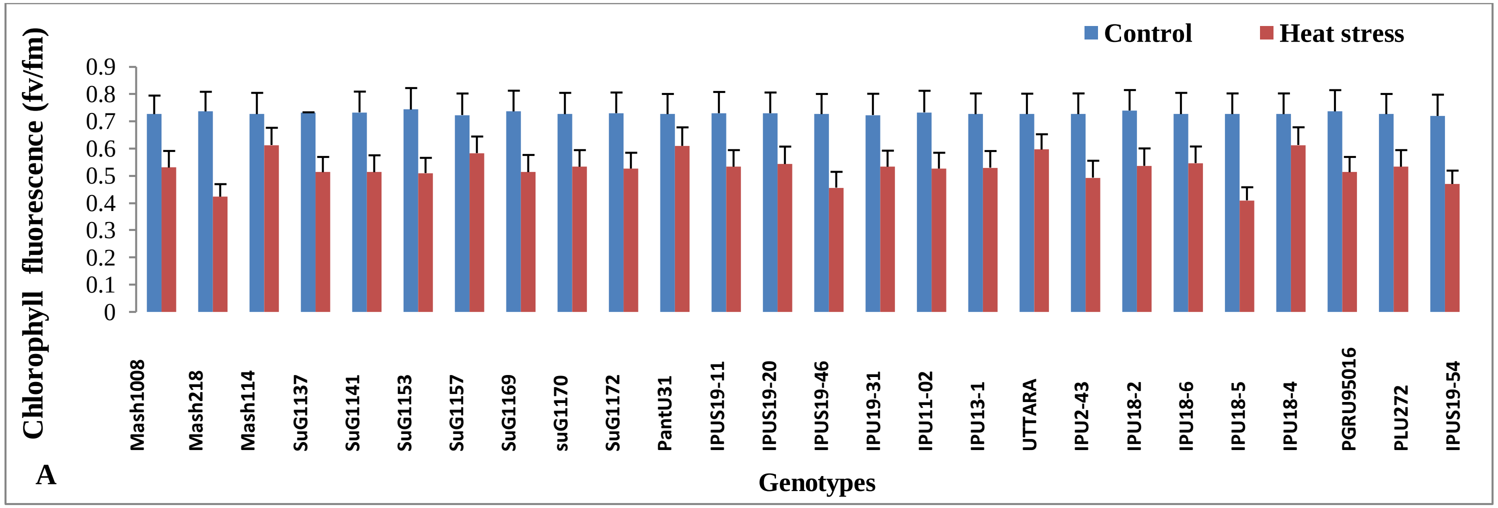

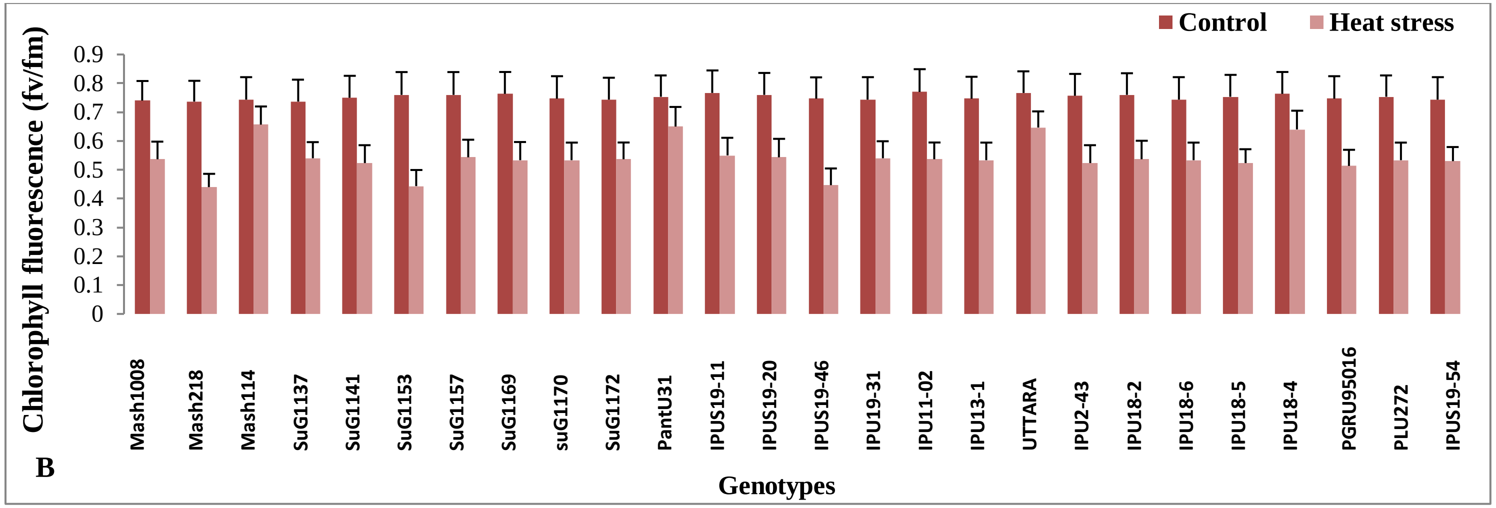

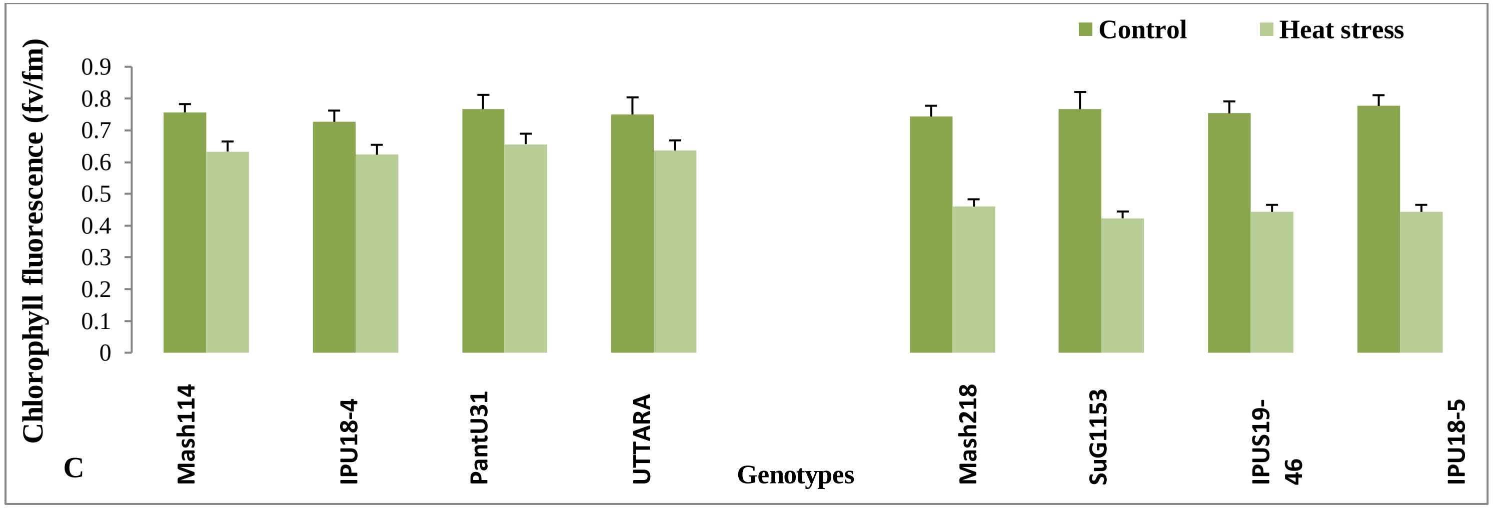

Supplementary Fig.S5: Chlorophyll fluorescence of Urdbean genotypes under control (normal-sown; Control) and heat stress environment during 2018 (A), 2019 (B) and in controlled environment of growth chamber (C; GC). LSD values (P < 0.05); genotype × treatment: 0.13 (2018), 0.16 (2019), 0.11 (GC). Values are means + SE. (n = 3).

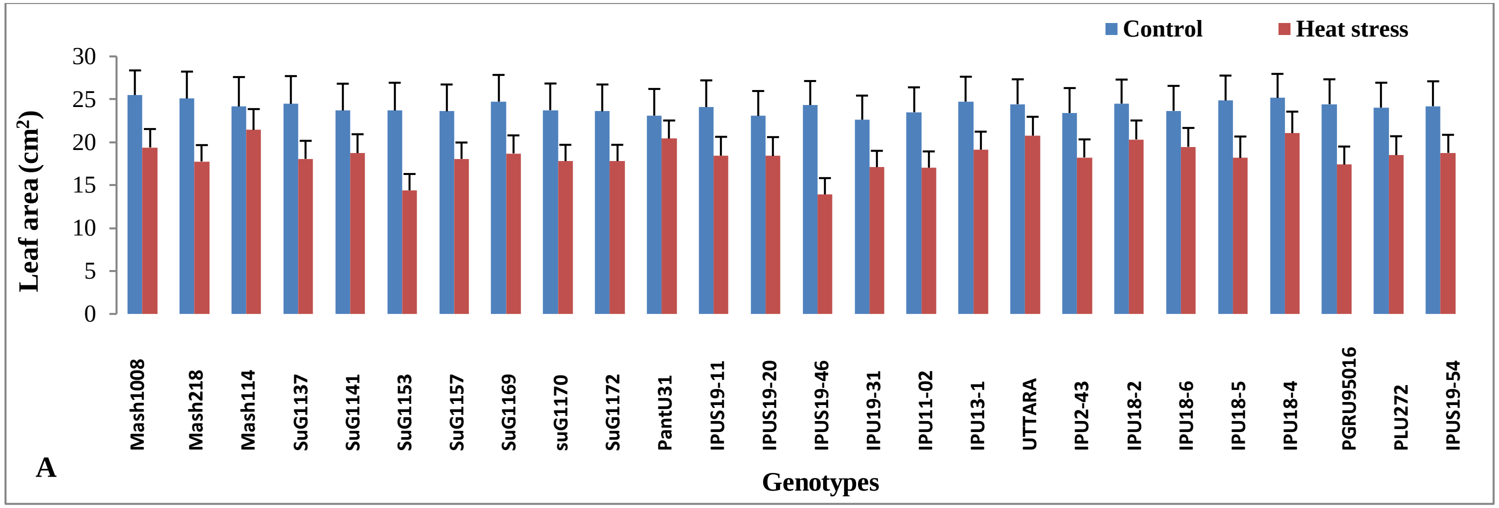
Control
Heat stress
Leaf area (cm2)
30
25
20
15
10
5
0
Mash1008
Mash218
Mash114
SuG1137
SuG1141
SuG1153
SuG1157
SuG1169
suG1170
SuG1172
PantU31
IPUS19-11
IPUS19-20
IPUS19-46
IPU19-31
IPU11-02
IPU13-1
UTTARA
IPU2-43
IPU18-2
IPU18-6
IPU18-5
IPU18-4
PGRU95016
PLU272
IPUS19-54
A
Genotypes

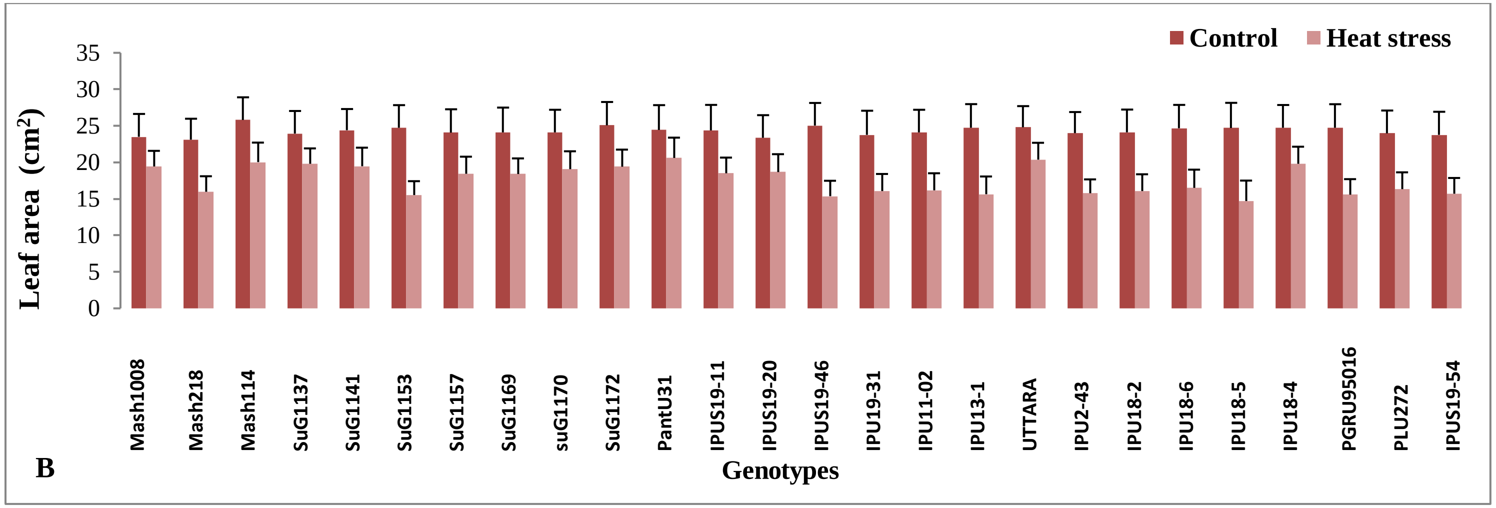
Control
Heat stress
Leaf area (cm2)
35
30
25
20
15
10
5
0
Mash1008
Mash218
Mash114
SuG1137
SuG1141
SuG1153
SuG1157
SuG1169
suG1170
SuG1172
PantU31
IPUS19-11
IPUS19-20
IPUS19-46
IPU19-31
IPU11-02
IPU13-1
UTTARA
IPU2-43
IPU18-2
IPU18-6
IPU18-5
IPU18-4
PGRU95016
PLU272
IPUS19-54
B
Genotypes

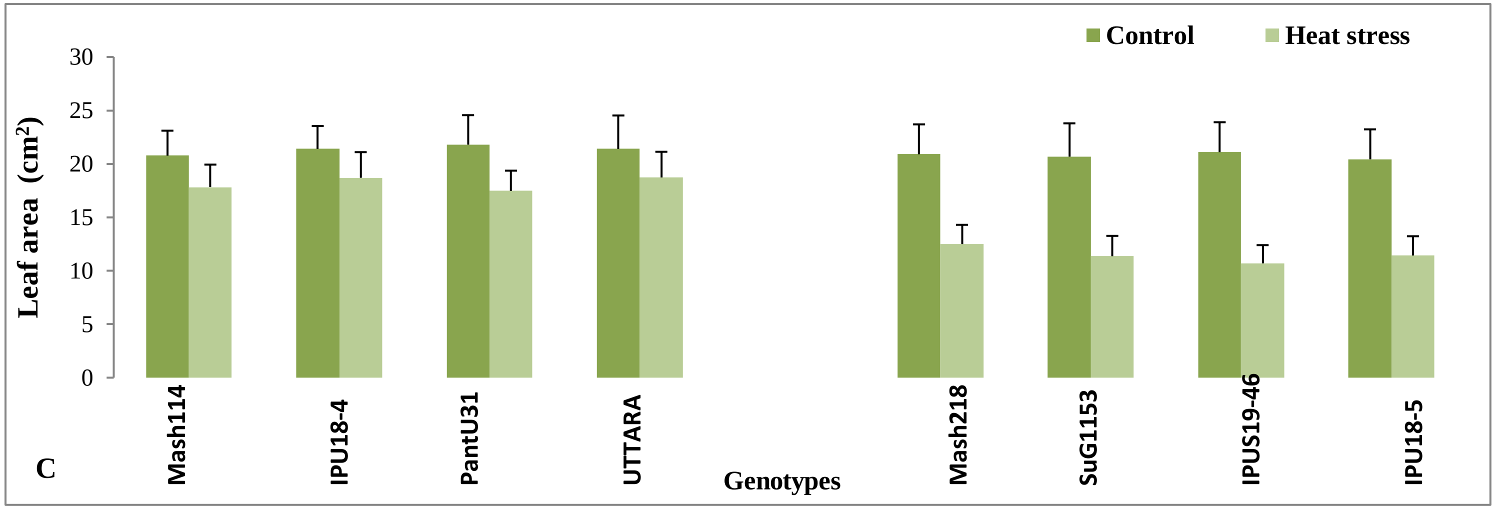

Supplementary Fig.S6: Leaf area of Urdbean genotypes under control (normal-sown; Control) and heat stress environment during 2018 (A),2019 (B) and in controlled environment of growth chamber (C; GC). LSD values ($P < 0.05$); genotype × treatment: 3.8 (2018), 3.3 (2019), 3.5 (GC). Values are means + SE. ($n = 3$).

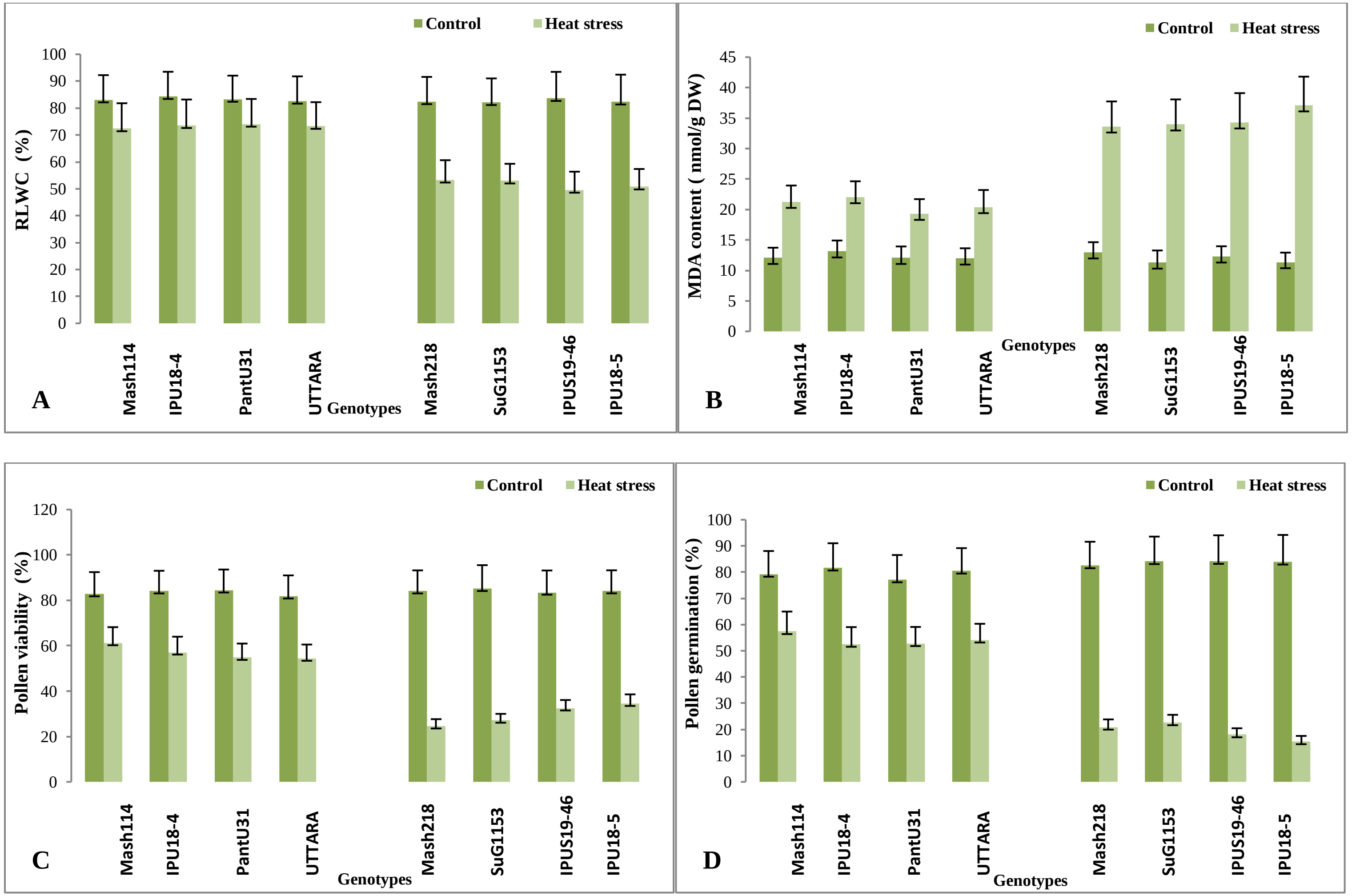

Supplementary Fig.S7: Relative leaf water content (RLWC; A), Malondialdehyde (MDA; B), Pollen viability % (PV; C) and Pollen germination % (PG; D) of Urdbean genotypes under control (normal-sown; Control) and heat stress environment during 2018 (A), 2019 (B) and in controlled environment of growth chamber (C; GC). LSD values ($P < 0.05$); genotype × treatment: 3.15 (RLWC), 5.4 (MDA), 7.8 (PV), 8.3 (PG). Values are means + SE. ($n = 3$).
